# Supplementary material for: Barbarigenesis and the collapse of complex societies: Rome and after
Source: PLoS One. 2021 Sep 16;16(9):e0254240. doi: 10.1371/journal.pone.0254240 (PMC8445445; doi:10.1371/journal.pone.0254240)

In[1]:= (* This notebook shows outcomes for the two person game given by Equations 1 to 4. It is the basis for Figures 1 and 8. *)

In[2]:= (* Variables are
r1 and r2 are the resources of each player
f1 and f2 are the fighting effort of each player
r1-f1 and r2-f2 is the production of each player
m is the decisiveness of conflict *)

In[3]:= (* Each player tries to maximize his income

$\frac{f1^m}{f1^m+f2^m}(r1-f1+r2-f2)$ and $\frac{f2^m}{f1^m+f2^m}(r1-f1+r2-f2)$

subject to the constraints

$f1-s01^2>=0$ and $f2-s02^2>=0$ (fighting effort is nonnegative)

$r1-f1-sr1^2>=0$ and $r2-f2-sr2^2>0$ (production is nonnegative) *)

In[4]:= Clear[r1, r2, f1, f2, m, opt1, opt2, constraintlist, initiallist]

In[5]:= (* We take the first derivative of income (shown for player 1) *)

In[6]:= $D\left[f1^m/(f1^m+f2^m)\,(r1-f1+r2-f2),\ f1\right]$

Out[6]= $$-\frac{f1^m}{f1^m+f2^m}-\frac{f1^{-1+2m}\,m\,(-f1-f2+r1+r2)}{(f1^m+f2^m)^2}+\frac{f1^{-1+m}\,m\,(-f1-f2+r1+r2)}{f1^m+f2^m}$$

In[7]:= (* Rearranging terms and adding Lagrange multipliers to handle our constraints we get: *)

In[8]:= $$opt1 := m\frac{f1^{m-1}}{f1^m+f2^m}(r1-f1+r2-f2)-m\frac{f1^{2m-1}}{(f1^m+f2^m)^2}(r1-f1+r2-f2)-\frac{f1^m}{f1^m+f2^m}+\lambda r1+\lambda 01$$

In[9]:= $$opt2 := m\frac{f2^{m-1}}{f1^m+f2^m}(r1-f1+r2-f2)-m\frac{f2^{2m-1}}{(f1^m+f2^m)^2}(r1-f1+r2-f2)-\frac{f2^m}{f1^m+f2^m}+\lambda r2+\lambda 02$$

In[10]:= (* We set the first derivative above equal to zero,
and satisfy the constraints below *)

In[11]:= constraintlist :=
$\{r1-f1-sr1^2,\ f1-s01^2,\ \lambda r1\,sr1,\ \lambda 01\,s01,\ r2-f2-sr2^2,\ f2-s02^2,\ \lambda r2\,sr2,\ \lambda 02\,s02\}$

In[12]:= (* Putting these together,
below is the list of conditions to be satisfied for a given r1 r2 and m. *)

In[13]:= Join[{opt1, opt2}, constraintlist]

Out[13]= $$\left\{-\frac{f1^m}{f1^m+f2^m}-\frac{f1^{-1+2m}\,m\,(-f1-f2+r1+r2)}{(f1^m+f2^m)^2}+\frac{f1^{-1+m}\,m\,(-f1-f2+r1+r2)}{f1^m+f2^m}+\lambda 01+\lambda r1,\right.$$
$$-\frac{f2^m}{f1^m+f2^m}-\frac{f2^{-1+2m}\,m\,(-f1-f2+r1+r2)}{(f1^m+f2^m)^2}+\frac{f2^{-1+m}\,m\,(-f1-f2+r1+r2)}{f1^m+f2^m}+\lambda 02+\lambda r2,$$
$$\left.-f1+r1-sr1^2,\ f1-s01^2,\ sr1\,\lambda r1,\ s01\,\lambda 01,\ -f2+r2-sr2^2,\ f2-s02^2,\ sr2\,\lambda r2,\ s02\,\lambda 02\right\}$$

In[14]:= (* We need to give the FindRoot finction some initial values to work with *)

```
In[15]:= initiallist := Transpose[{{f1, f2, λr1, λ01, λr2, λ02, sr1, s01, sr2, s02},
            {4, 4, -.1, -.1, -.1, -.1, 1, 1, 1, 1}}]

In[16]:= (* Here's the result for r1=
          10 r2=5 m=1. The two players put equal effort into fighting *)

In[17]:= FindRoot[Join[{opt1, opt2}, constraintlist] /. {r1 → 10, r2 → 5, m → 1}, initiallist]

Out[17]= {f1 → 3.75, f2 → 3.75, λr1 → -4.66983 × 10^-21, λ01 → 1.54911 × 10^-22, λr2 → -2.48058 × 10^-22,
          λ02 → -1.85817 × 10^-24, sr1 → 2.5, s01 → 1.93649, sr2 → 1.11803, s02 → 1.93649}

In[18]:= (* Here's the result for r1=
          10 r2=3 m=1. We get a corner solution for player 2 who
             is putting all his resources into fighting. *)

In[19]:= FindRoot[Join[{opt1, opt2}, constraintlist] /. {r1 → 10, r2 → 3, m → 1}, initiallist]

Out[19]= {f1 → 3.245, f2 → 3., λr1 → -7.83002 × 10^-22, λ01 → 4.04598 × 10^-18, λr2 → -0.081666,
          λ02 → 1.40035 × 10^-20, sr1 → 2.59904, s01 → 1.80139, sr2 → 8.76941 × 10^-16, s02 → 1.73205}

In[20]:= (* We define the function empbarb[r1,r2,m]
          which gives the equlibrium income for each player. *)

In[21]:=
         empbarb[rr1_, rr2_, mm_] :=
          {(f1^m/(f1^m + f2^m) (r1 - f1 + r2 - f2)), (f2^m/(f1^m + f2^m) (r1 - f1 + r2 - f2))} /.
            FindRoot[Join[{opt1, opt2}, constraintlist] /. {r1 → rr1, r2 → rr2, m → mm},
             initiallist] /. {r1 → rr1, r2 → rr2, m → mm}

In[22]:= (* Here are players' incomes for r1=10 r2=3 m=1. *)

In[23]:= empbarb[10, 3, 1]

Out[23]= {3.51, 3.245}

In[24]:= (* Here is a plot of players' incomes when r1=
          10 and r2 goes from 0 to 10 and m=1 *)

In[25]:= (* FIGURE 1 *)
```

In[26]:=
```
ListLinePlot[Transpose[Join[{{10, 0}}, Table[empbarb[10, y, 1], {y, .2, 10, .2}]]],
 PlotRange → All, DataRange → {0, 10}, AxesLabel → {"player 2 resources", "income"},
 PlotLabels → {Callout["player 1, resources=10", {Scaled[0.45], Above}],
   Callout["player 2", {Scaled[0.25], Below}]}]
```

Out[26]=

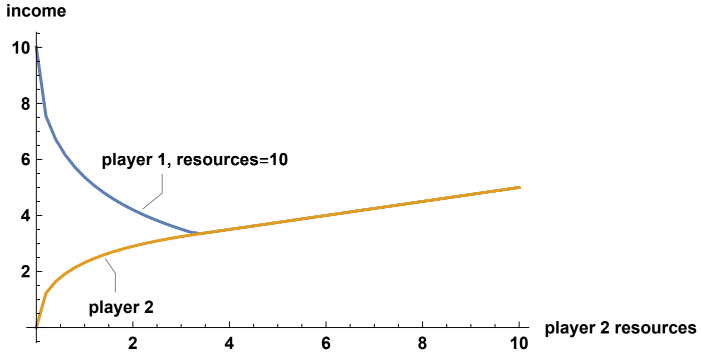

In[27]:=
```
(* "Ancient" war: here is a plot of players' incomes when r1=
 10 and r2 goes from 0 to 10 and m=1 *)
```

In[28]:=
```
(* FIGURE 8a *)
```

In[29]:=
```
ListLinePlot[
 {Transpose[Join[{{10, 0}}, Table[empbarb[10, y, 1], {y, .2, 10, .2}]]][[1]],
  Table[5, {y, 0, 10, .2}]}, PlotRange → All,
 DataRange → {0, 10}, AxesLabel → {"player 2 resources", "income"},
 PlotLabels → {Callout["player 1, resources=10", {Scaled[0.45], Above}]},
 PlotStyle → {, Dotted}]
```

Out[29]=

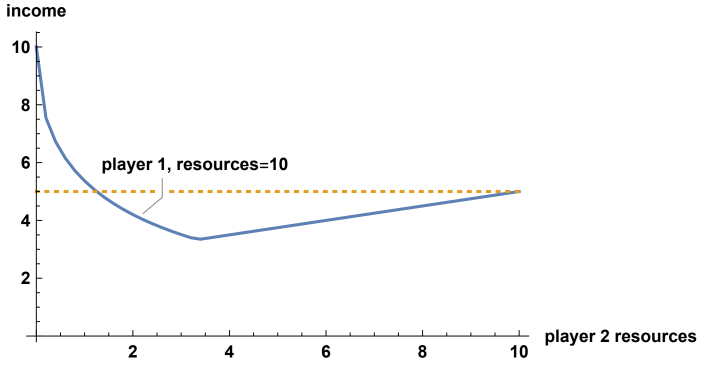

In[30]:=
```
(* "Modern" war: here is a plot of players' incomes when r1=
 10 and r2 goes from 0 to 10 and m=2 *)
```

In[31]:=
```
(* FIGURE 8b *)
```

In[32]:=
```
ListLinePlot[
 {Transpose[Join[{{10, 0}}, Table[empbarb[10, y, 2], {y, .2, 10, .2}]]][[1]],
  Table[3.3333333, {y, 0, 10, .2}]}, PlotRange → All,
 DataRange → {0, 10}, AxesLabel → {"player 2 resources", "income"},
 PlotLabels → {Callout["player 1, resources=10", {Scaled[0.45], Above}]},
 PlotStyle → {, Dotted}]
```

Out[32]=

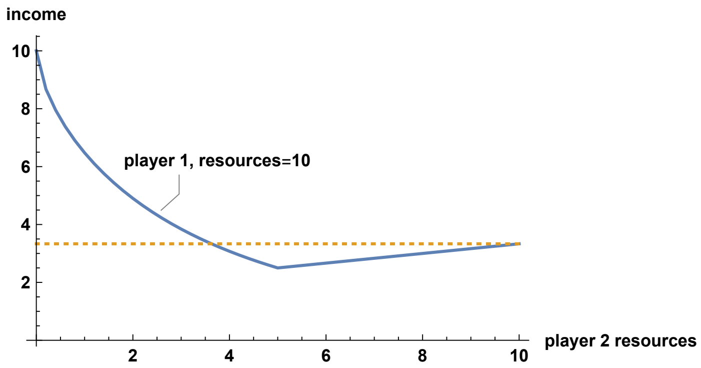

In[33]:=
```
(* Even more decisive: here is a plot of players' incomes when r1=
 10 and r2 goes from 0 to 10 and m=3 *)
```

In[34]:=
```
ListLinePlot[
 {Transpose[Join[{{10, 0}}, Table[empbarb[10, y, 3], {y, .2, 10, .2}]]][[1]],
  Table[2.5, {y, 0, 10, .2}]}, PlotRange → All,
 DataRange → {0, 10}, AxesLabel → {"player 2 resources", "income"},
 PlotLabels → {Callout["player 1, resources=10", {Scaled[0.45], Above}]},
 PlotStyle → {, Dotted}]
```

Out[34]=

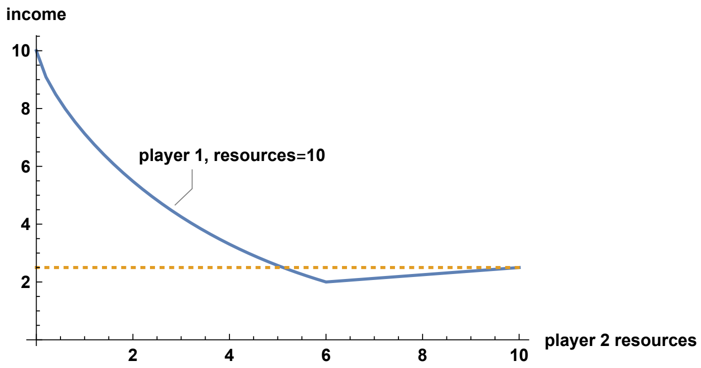

Supplement: S1 File — (PDF) [file pone.0254240.s001.pdf]
