# Supplementary material for: Barbarigenesis and the collapse of complex societies: Rome and after
Source: PLoS One. 2021 Sep 16;16(9):e0254240. doi: 10.1371/journal.pone.0254240 (PMC8445445; doi:10.1371/journal.pone.0254240)

```
In[206]:= (* In order to make this notebook work it is necessary to first
            run the notebook "barbarigen n player". The function whistory -
          wealth as a function of space and time - is defined there. *)

In[207]:= (* This notebook fits data to the model given in the notebook
          "barbarigen n player". It is the basis for Figures 7 5 and 6. *)

In[208]:= (* OUTLINE
          PART ONE. Ascribe spatial positions to each country and show observed
          versus expected population density over time for each. Figure 7
          PART TWO. Compare ascribed versus geogrphic distances. Figure 5
          PART THREE. Ascrtibed distance versus imperial history. Figure 6
          *)

In[209]:= (* PART ONE. Ascribe spatial positions to each country and show
          observed versus expected population density over time for each. *)

In[210]:= Clear[begenesisw, countrylist, countrypops, countrydistances,
          density, year, weightyear, sumsq, initialdistance, fitwealth,
          regr, int, assigndistance, countryfigure, countrylistranked]

In[211]:= (* The function begnesisw is a contunuous function giving wealth in space and
          time with time from 0 to 1200 CE and space in arbitrary units from 0 to 100. *)

In[212]:= bgenesisw = ListInterpolation[whistory, {{0, 1200}, {0, 100}}]

Out[212]= InterpolatingFunction[ Domain: {{0., 1.20×10^3}, {0., 100.}}
                                 Output: scalar ]

In[213]:= countrylist := {England, Ireland, Scandinavia, France, Belgium,
            Netherlands, Germany, Poland, Russia, Czechoslovakia, Austria,
            Hungary, Romania, Spain, Portugal, Italy, Yugoslavia, Greece, Bulgaria}

In[214]:= (* List of country populations in millions from -
          199 to 1300 CE from McEvedy and Jones. Last number is area in million km sq *)

In[215]:= (* Notes regarding the data. Interpolation was used to fill
            in missing data for Ireland (3 points) Netherlands (3 points)
            Yugoslavia (4 points) Greece (3 points) Bulgaria (4 points)
            Scotland Switzerland Turkey-in-Europe Albania and Cyprus
            and other islands were omitted. Not enough estimates.
             England includes Wales.
             Belgium includes Luxembourg.
             Russia is Soviet Union in Europe. *)
```

```
In[216]:= englandpop := {0.4, 0.6, 0.7, 0.8, 0.6, 0.8, 1.5, 1.75, 2.5, 3.75, 0.15};
irelandpop := {0.1, 0.1, 0.15, 0.2, 0.233, 0.267, 0.3, 0.4, 0.6, 0.8, 0.08};
scandinaviapop := {0.4, 0.5, 0.6, 0.6, 0.7, 0.8, 1, 1.25, 1.5, 1.75, 1.15};
francepop := {4, 5, 6.5, 5, 4.5, 5, 6.5, 7.75, 10.5, 16, 0.55};
belgiumpop := { 0.3, 0.3, 0.4, 0.3, 0.3, 0.3, 0.4, 0.6, 0.9, 1.25, 0.03};
netherlandspop := {0.2, 0.2, 0.2, 0.2, 0.2, 0.2, 0.3, 0.4, 0.6, 0.8, 0.03};
germanypop := { 2, 3, 3.5, 3.5, 3, 3.25, 3.54, 4, 6, 9, 0.36};
polandpop := {0.3, 0.5, 0.6, 0.8, 0.6, 1, 1.25, 1.5, 2.25, 3.5, 0.31};
russiapop := {1.75, 2, 2.5, 3, 3, 3.5, 4, 6, 9, 9, 4.77};
czechoslovakiapop := {0.8, 1, 1.25, 1, 0.7, 1, 1.25, 1.5, 2, 3, 0.13};
austriapop := {0.3, 0.5, 0.6, 0.5, 0.4, 0.5, 0.7, 0.9, 1.25, 2, 0.08};
hungarypop := {0.3, 0.3, 0.3, 0.3, 0.2, 0.3, 0.5, 0.6, 0.8, 1.25, 0.09};
romaniapop := {0.6, 0.8, 0.8, 0.6, 0.5, 0.6, 0.8, 0.9, 1, 1.5, 0.24};
spainpop := {4, 4.5, 5, 4.5, 3.5, 3.75, 4, 4.5, 5.5, 7.5, 0.5};
portugalpop := {0.4, 0.5, 0.5, 0.5, 0.4, 0.4, 0.6, 0.7, 0.9, 1.25, 0.09};
italypop := {5, 7, 7, 5, 3.5, 4, 5, 5.75, 7.25, 10, 0.3};
yugoslaviapop := { 1.25, 1.5, 1.75, 1.5, 1.25, 1.5, 1.75, 1.91, 2.08, 2.25, 0.26};
greecepop := {2.5, 2, 2, 1.5, 0.8, 0.9, 1, 1.08, 1.17, 1.25, 0.13};
bulgariapop := { 0.5, 0.5, 0.8, 0.65, 0.5, 0.65, 0.8, 0.86, 0.93, 1, 0.11};
europepop :=
 {25.1, 30.8, 35.15, 30.45, 24.883, 28.717, 35.19, 42.35, 56.73, 76.85, 9.36}

In[236]:= countrypops := {englandpop, irelandpop, scandinaviapop, francepop,
   belgiumpop, netherlandspop, germanypop, polandpop, russiapop,
   czechoslovakiapop, austriapop, hungarypop, romaniapop , spainpop,
   portugalpop, italypop, yugoslaviapop, greecepop, bulgariapop}

In[237]:= (* countrydistances will be assigned to fit the model *)

In[238]:= countrydistances := {englandx, irelandx, scandinaviax, francex, belgiumx,
   netherlandsx, germanyx, polandx, russiax, czechoslovakiax, austriax, hungaryx,
   romaniax, spainx, portugalx, italyx, yugoslaviax, greecex, bulgariax}

In[239]:= (* The function density[place] gives the population
 density of a place over time from 0 CE to 1200 CE *)

In[240]:= density[pl_] := Drop[Drop[pl, -2], 1] / Last[pl]

In[241]:= Variance[Flatten[Map[density, countrypops]]]

Out[241]= 28.501

In[242]:= % * 19 * 8

Out[242]= 4332.15

In[243]:= (* Because McEvedy and Jones give estimates every second
 century up to 1000 and every century after 1000 we assign the
 centuries 1100 and 1200 half the weight when fitting the data. *)
```

```
In[244]:= year := {0, 200, 400, 600, 800, 1000, 1100, 1200}

In[245]:= weightyear := {1, 1, 1, 1, 1, 1, .5, .5}

In[246]:= (* The function sumsq[pl,plx,reg,intercept] gives the squared difference between
          estmated population density and the assigned population density for a
          given ascribed spatial distance and regression multiplier and intercept. *)

In[247]:= sumsq[pl_, plx_, reg_, intercept_] :=
          SquaredEuclideanDistance[Flatten[Thread[bgenesisw[year, plx]]] * reg * weightyear,
           density[pl] * weightyear - intercept]

In[248]:= (* We can fit the model for one country at a time. *)

In[249]:= FindMinimum[sumsq[italypop, x, reg, int], {{x, 10}, {reg, 50}, {int, 10}}]

Out[249]= {3.47963, {x → 6.49378, reg → 10.8883, int → 1.70236}}

In[250]:= (* But what we really want to do is fit all the countries simultaneously
          ascribing a diatance to each and a single regression multiplier and intercept
          for all. We first give Mathmeatica's FindMinimum function some intial values. *)

In[251]:= initialdistance = {{englandx, 38}, {irelandx, 87}, {scandinaviax, 99}, {francex, 11},
           {belgiumx, 8}, {netherlandsx, 23}, {germanyx, 15}, {polandx, 87}, {russiax, 99},
           {czechoslovakiax, 17}, {austriax, 24}, {hungaryx, 79}, {romaniax, 95}, {spainx, 15},
           {portugalx, 39}, {italyx, .1}, {yugoslaviax, 31}, {greecex, 3}, {bulgariax, 31}}

Out[251]= {{englandx, 38}, {irelandx, 87}, {scandinaviax, 99}, {francex, 11},
          {belgiumx, 8}, {netherlandsx, 23}, {germanyx, 15}, {polandx, 87}, {russiax, 99},
          {czechoslovakiax, 17}, {austriax, 24}, {hungaryx, 79}, {romaniax, 95}, {spainx, 15},
          {portugalx, 39}, {italyx, 0.1}, {yugoslaviax, 31}, {greecex, 3}, {bulgariax, 31}}

In[252]:= (* The first number that the function fitwealth gives is
            the total sum of squares for all the countries. We used trial-
           and-error to find the combination of r and a and c that minimizes
            this number. We constrain the function FindMinimum to
            find values less than 100 for ascribed distance.) *)

In[253]:= fitwealth = FindMinimum[
           {Total[MapThread[sumsq, {countrypops, countrydistances, ConstantArray[reg, 19],
               ConstantArray[int, 19]}]], irelandx < 100, francex > 10, belgiumx > 0,
            netherlandsx > 0, italy > 0, germanyx > 0, scandinaviax < 100, russiax < 100},
           Join[initialdistance, {{reg, 5}, {int, 3}}]]

Out[253]= FindMinimum[
          {Total[MapThread[sumsq, {countrypops, countrydistances, ConstantArray[reg, 19],
              ConstantArray[int, 19]}]], irelandx < 100, francex > 10, belgiumx > 0,
           netherlandsx > 0, italy > 0, germanyx > 0, scandinaviax < 100, russiax < 100},
          Join[initialdistance, {{reg, 5}, {int, 3}}]]
```

In[254]:=
```
fitwealth = FindMinimum[
  {Total[MapThread[sumsq, {countrypops, countrydistances, ConstantArray[reg, 19],
      ConstantArray[int, 19]}]], scandinaviax < 100,
   russiax < 100}, Join[initialdistance, {{reg, 5}, {int, 3}}]]
```

InterpolatingFunction: Input value {0, −6.23329} lies outside the range of data in the interpolating function. Extrapolation will be used.

InterpolatingFunction: Input value {200, −6.23329} lies outside the range of data in the interpolating function. Extrapolation will be used.

InterpolatingFunction: Input value {400, −6.23329} lies outside the range of data in the interpolating function. Extrapolation will be used.

General: Further output of InterpolatingFunction::dmval will be suppressed during this calculation.

Out[254]=
```
{492.44, {englandx → 45.2412, irelandx → 84.5601, scandinaviax → 100.,
  francex → 16.3223, belgiumx → 12.0372, netherlandsx → 30.3695,
  germanyx → 20.9474, polandx → 84.9489, russiax → 100., czechoslovakiax → 23.649,
  austriax → 31.5772, hungaryx → 77.5424, romaniax → 92.4035, spainx → 21.4085,
  portugalx → 43.9487, italyx → -1.10052, yugoslaviax → 38.8418,
  greecex → 7.28061, bulgariax → 38.682, reg → 7.21449, int → 1.13591}}
```

In[255]:=
```
fitwealth = FindMinimum[
  {Total[MapThread[sumsq, {countrypops, countrydistances, ConstantArray[reg, 19],
      ConstantArray[int, 19]}]]}, Join[initialdistance, {{reg, 5}, {int, 3}}]]
```

InterpolatingFunction: Input value {0, −3.06472} lies outside the range of data in the interpolating function. Extrapolation will be used.

InterpolatingFunction: Input value {0, 136.571} lies outside the range of data in the interpolating function. Extrapolation will be used.

InterpolatingFunction: Input value {0, 139.016} lies outside the range of data in the interpolating function. Extrapolation will be used.

General: Further output of InterpolatingFunction::dmval will be suppressed during this calculation.

Out[255]=
```
{486.246, {englandx → 45.8612, irelandx → 85.1408, scandinaviax → 111.197,
  francex → 16.3694, belgiumx → 12.0424, netherlandsx → 30.6092, germanyx → 21.0448,
  polandx → 85.5328, russiax → 111.41, czechoslovakiax → 23.7757,
  austriax → 31.8135, hungaryx → 78.0261, romaniax → 93.3004, spainx → 21.5092,
  portugalx → 44.5791, italyx → -1.15518, yugoslaviax → 39.3011,
  greecex → 7.2503, bulgariax → 39.0915, reg → 7.15859, int → 1.21824}}
```

In[256]:=
```
fitwealth[[1]] / 19 / 8
```

Out[256]=
```
3.19899
```

In[257]:=
```
fitwealth[[1]] / 19 / 8 / Variance[Flatten[Map[density, countrypops]]]
```

Out[257]=
```
0.112241
```

In[258]:=
```
(* Given the regression multiplier and intercept and
 ascribed distance for a country we can plot the predicted
 population density for that country. In this case Italy *)
```

```
In[259]:= regr := reg /. fitwealth[[2]]; intr := int /. fitwealth[[2]]

In[260]:= assigndistance[nameacountry_] :=
           countrydistances[[First[Position[countrylist, nameacountry]]]] /. fitwealth[[2]]

In[261]:= countryname := Italy

In[262]:= Plot[bgenesisw[t, assigndistance[countryname]] * regr + intr,
           {t, 0, 1200}, PlotRange → {0, 30}, PlotStyle → Red]
```

InterpolatingFunction: Input value {0.0245143, −1.15518} lies outside the range of data in the interpolating function. Extrapolation will be used.

Out[262]=

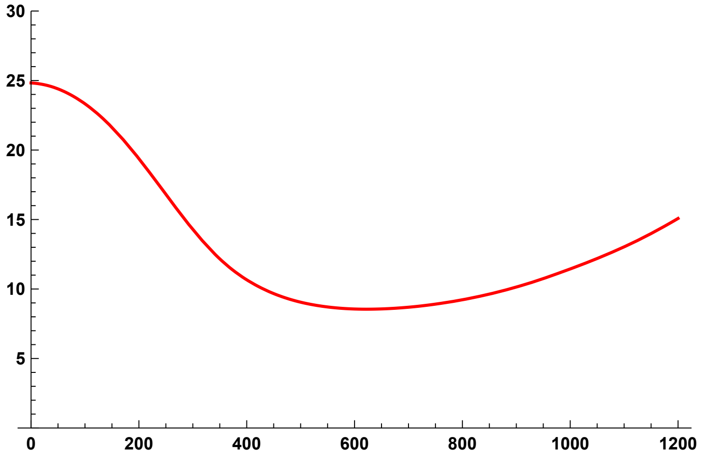


```
In[263]:= Plot[bgenesisw[t, assigndistance[countryname]] * regr + intr,
           {t, 0, 1200}, PlotRange → {0, 30}, PlotStyle → Red,
           Ticks → {{0, 400, 800, 1200}, {5, 10, 15, 20, 25, 30}}]
```

InterpolatingFunction: Input value {0.0245143, −1.15518} lies outside the range of data in the interpolating function. Extrapolation will be used.

Out[263]=

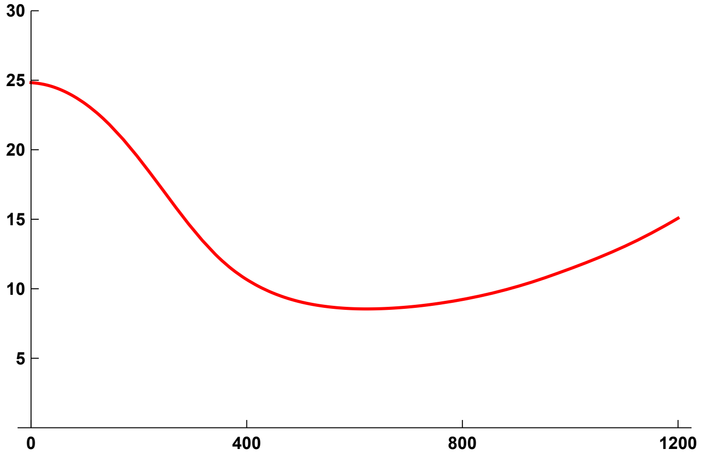


```
In[264]:= (* We can also plot the estimated population density from McEvedy and Jones *)
```

In[265]:=
```
ListLinePlot[Transpose[{year,
   density[First[countrypops[[First[Position[countrylist, countryname]]]]]]}],
 PlotRange → {0, 30}, PlotMarkers -> {Automatic, 10}, PlotStyle → {Dashed, Black}]
```

Out[265]=

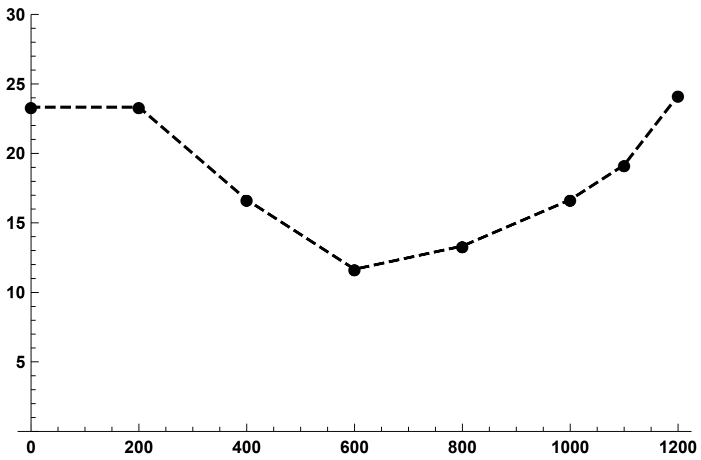

In[266]:=
```
(* We can compare predicted and estimated for Italy. *)
```

In[267]:=
```
countryfigure[nameacountry_] :=
 Show[Plot[bgenesisw[t, assigndistance[nameacountry]] * regr + intr, {t, 0, 1200},
   PlotRange → {0, 30}, PlotStyle → Red], ListLinePlot[Transpose[{year,
     density[First[countrypops[[First[Position[countrylist, nameacountry]]]]]]}],
   PlotRange → {0, 30}, PlotMarkers -> {Automatic, 10}, PlotStyle → {Dashed, Black}],
  PlotLabel → nameacountry, LabelStyle → {Larger, Bold},
  Ticks → {{{0, "0"}, {200, ""}, {400, "400"}, {600, ""},
     {800, "800"}, {1000, ""}, {1200, "1200"}}, {5, 10, 15, 20, 25, 30}}]
```

In[268]:=
```
countryfigure[Italy]
```

InterpolatingFunction: Input value {0.0245143, -1.15518} lies outside the range of data in the interpolating function. Extrapolation will be used.

Out[268]=

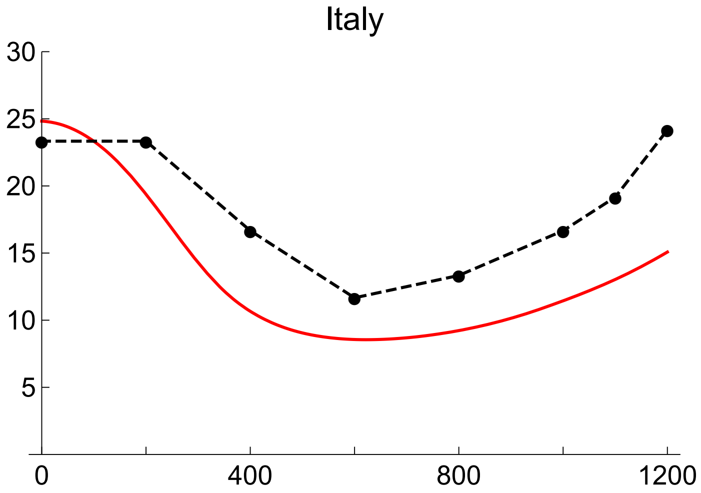

In[269]:=
```
(* Here is predicted and estimated for all the countries *)
```

In[270]:=
```
countrylistranked := Transpose[
    Sort[Transpose[Join[{countrylist, countrydistances /. fitwealth[[2]]}, 2]],
     #1[[2]] < #2[[2]] &]][[1]]
```

In[271]:=
```
(* Figure 7 *)
```

In[272]:=
```
Map[countryfigure, countrylistranked]
```

InterpolatingFunction: Input value {0.0245143, –1.15518} lies outside the range of data in the interpolating function. Extrapolation will be used.

InterpolatingFunction: Input value {0.0245143, 111.197} lies outside the range of data in the interpolating function. Extrapolation will be used.

InterpolatingFunction: Input value {0.0245143, 111.41} lies outside the range of data in the interpolating function. Extrapolation will be used.

General: Further output of InterpolatingFunction::dmval will be suppressed during this calculation.

Out[272]=

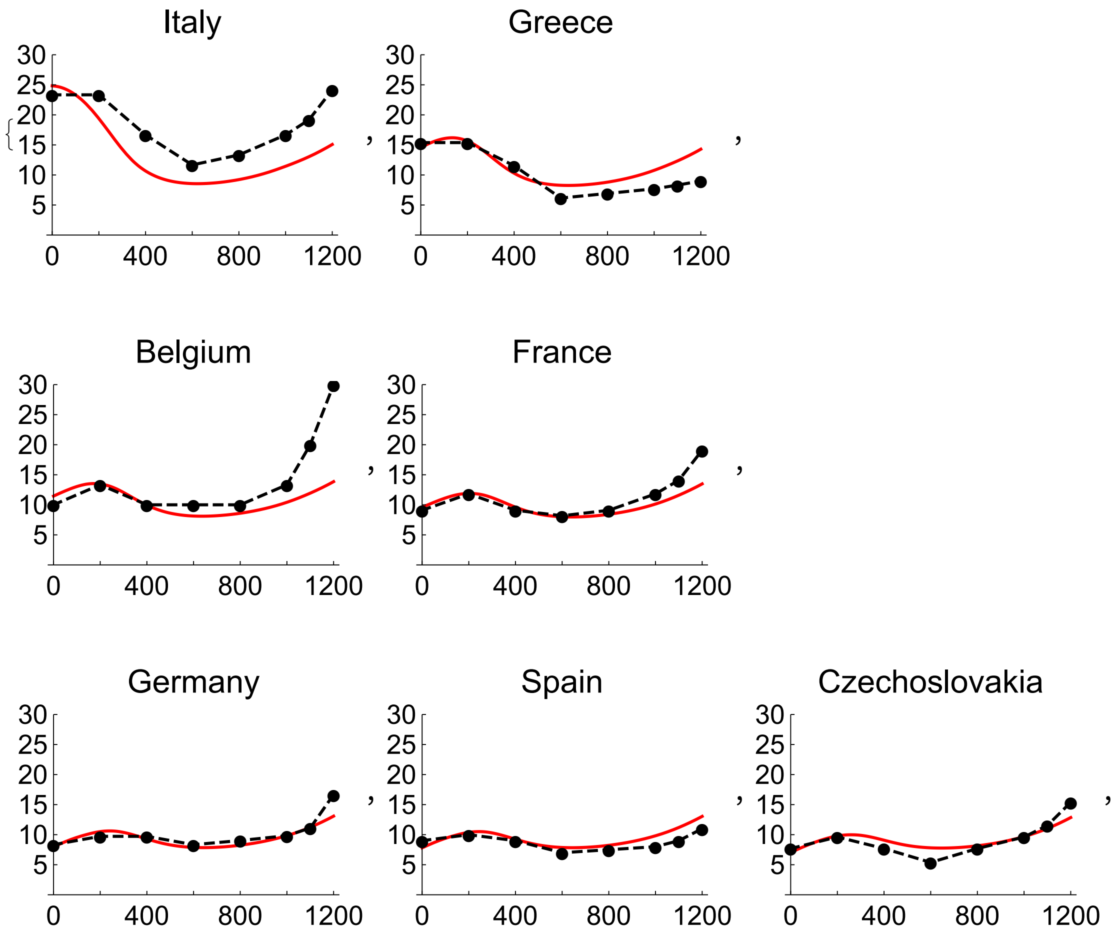

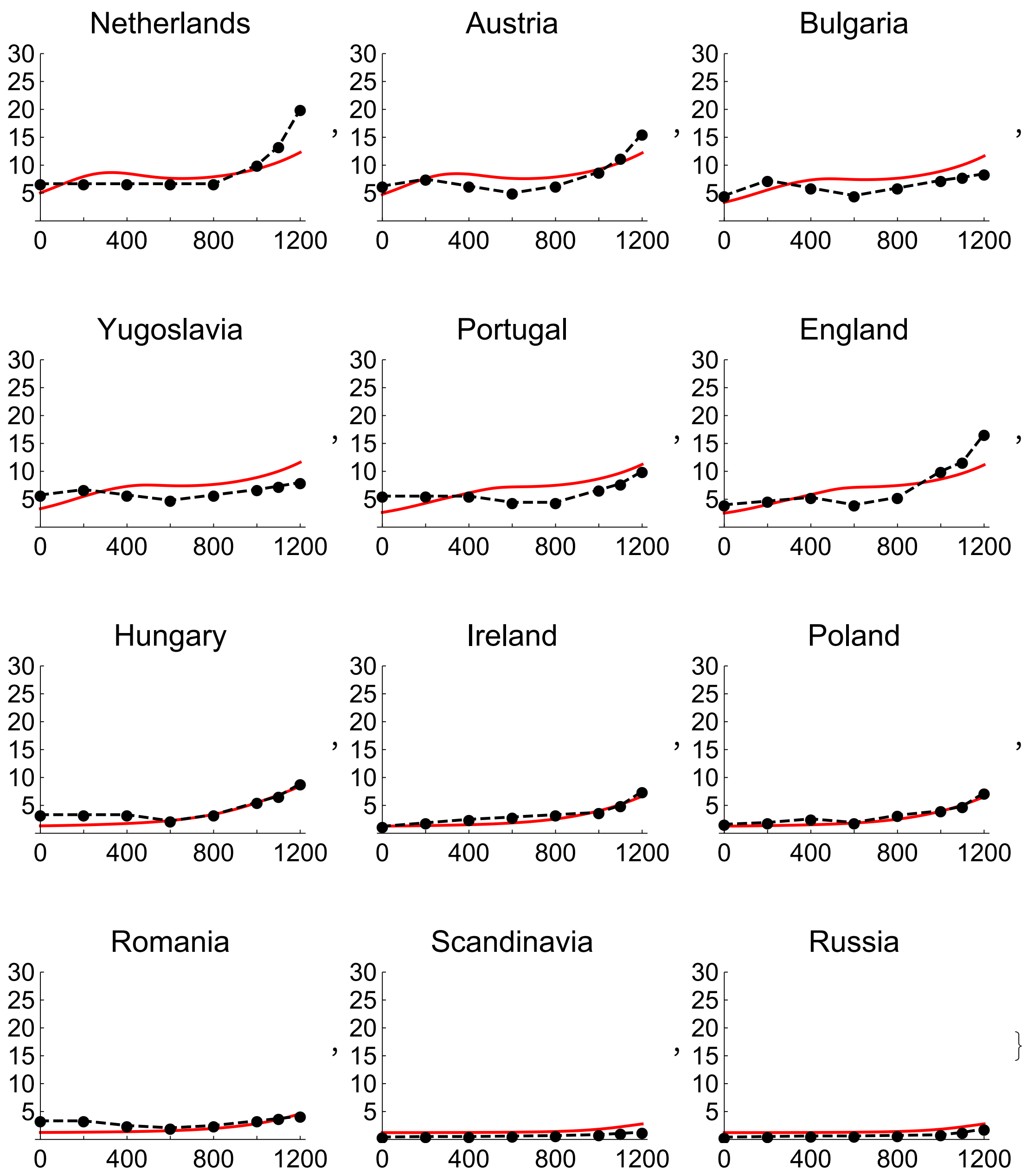

In[273]:= (* PART TWO. Compare ascribed versus geographic distances. *)

In[274]:= (* countrydeg gives the direction between
Rome and the center of the country in degrees. *)

```
In[275]:= englanddeg := 120; irelanddeg := 125; scandinaviadeg := 80;
          francedeg := 130; belgiumdeg := 110; netherlandsdeg := 105;
          germanydeg := 90;
          polanddeg := 55;
          russiadeg := 40;
          czechoslovakiadeg := 60;
          austriadeg := 60;
          hungarydeg := 40;
          romaniadeg := 15;
          spaindeg := 180;
          portugaldeg := 175;
          italydeg := 0;
          yugoslaviadeg := 25;
          greecedeg := -20;
          bulgariadeg := 0

In[276]:= countrydeg := {englanddeg, irelanddeg, scandinaviadeg,
            francedeg, belgiumdeg, netherlandsdeg, germanydeg, polanddeg,
            russiadeg, czechoslovakiadeg, austriadeg, hungarydeg, romaniadeg,
            spaindeg, portugaldeg, italydeg, yugoslaviadeg, greecedeg, bulgariadeg}

In[277]:= (* The chart below shows (1) the geographic direction of each
           country in degrees from Rome and (2) the ascribed distance of
           each country shown as distance from Italy in arbitrary units. *)

In[278]:= empiredistancemap := ListPlot[
            Transpose[{N[(countrydistances /. fitwealth[[2]]) * Cos[countrydeg Degree]],
               N[(countrydistances /. fitwealth[[2]]) * Sin[countrydeg Degree]]}] →
             {"England", "Ireland", "Scandinavia", "France", "Bg", "Netherlands",
              "Gm", "Poland", "Russia", "Cz", "Austria", "Hungary", "Romania",
              "Spain", "Portugal", "Italy", "Yugoslavia", "Greece", "Bulgaria"},
            AxesLabel → {"E/W distance", "N/S distance"}]

In[279]:= (* FIGURE 5 *)
```

In[280]:= empiredistancemap

Out[280]=

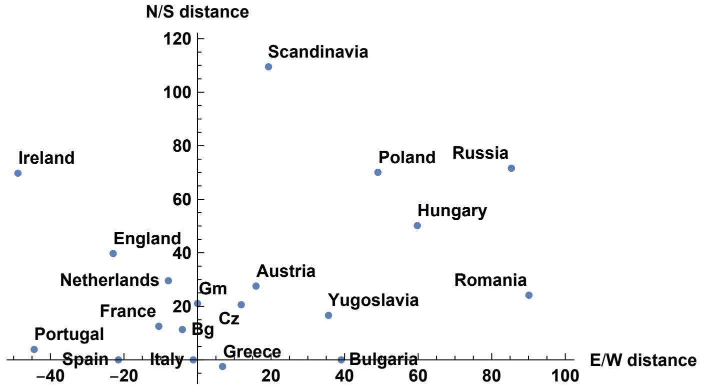

In[281]:= (* PART THREE. Ascrtibed distance versus imperial history. *)

In[282]:= (* Most of the dates below on the y axis are dates at which more than half the territory of the modern counry was incorporated into the Roman empire. For some countries that were only ever partially incorporated - Germany Hungary Romania - we give dates of partial incorporation. *)

In[283]:=
```
empireromebegin := ListPlot[
  {{englandx, 84} → "England", {francex, -44} → "France", {belgiumx, -44} → "Belgium",
    {germanyx, 85} → "Gm*", {austriax, 14} → "Austria", {hungaryx, 14} → "Hg*",
    {romaniax, 117} → "Rm*", {spainx, -64} → "Spain", {portugalx, -64} → "Portugal",
    {italyx, -240} → "Italy", {yugoslaviax, 14} → "Yugoslavia",
    {greecex, -148} → "Greece", {bulgariax, 54} → "Bulgaria"} /. fitwealth[[2]],
  PlotRange → {{-2, 120}, {-320, 210}}, AxesLabel → {distance, "year CE"},
  AxesOrigin → {-1, -320}, PlotStyle → Black]
```

In[284]:= empireromebegin

Out[284]=

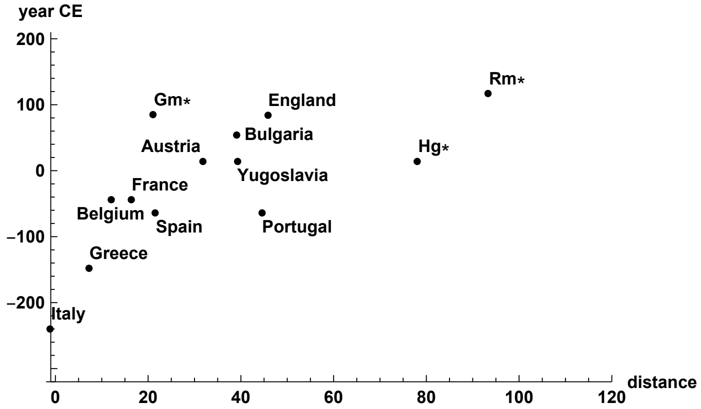

In[285]:= (* Another set of countries was never incorporated into the Roman empire or only partially incorporated. We assign these a nominal date of 200. *)

In[286]:=
```
empireromenone :=
 ListPlot[{{irelandx, 200} → "Ir", {netherlandsx, 200} → "Netherlands",
    {germanyx, 200} → "Gm*", {scandinaviax, 200} → "S/R",
    {russiax, 200}, {czechoslovakiax, 200} → "Cz",
    {hungaryx, 200} → "Hg*", {romaniax, 200} → "Rm*"} /. fitwealth[[2]],
  PlotRange → {{-2, 120}, {-320, 210}}, AxesLabel → {distance, "Year CE"},
  AxesOrigin → {-1, -320}, PlotStyle → Red]
```

In[287]:=
```
empireromenone
```

Out[287]=

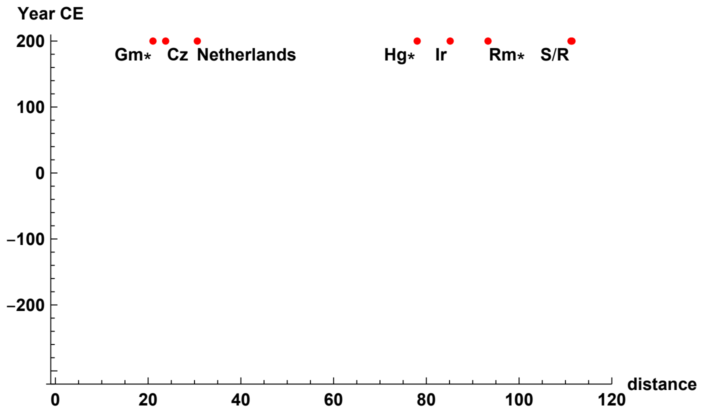

In[288]:=
```
(* We combine data to show how dates of incorporation
 or partial incorporation correspond to ascribed distance. *)
```

In[289]:=
```
Show[empireromebegin, empireromenone]
```

Out[289]=

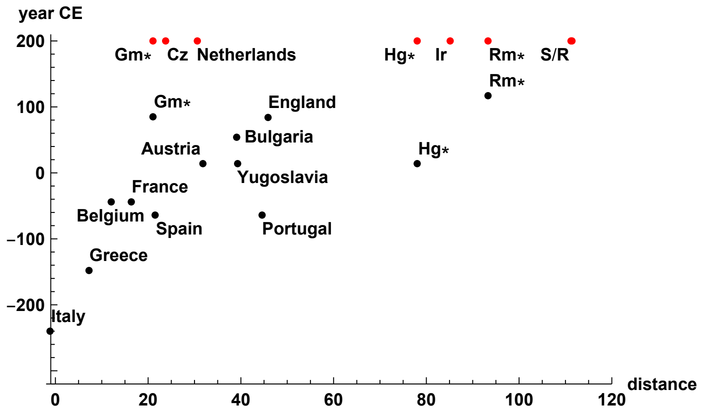

Supplement: S2 File — (PDF) [file pone.0254240.s002.pdf]
