# Supplementary material for: Barbarigenesis and the collapse of complex societies: Rome and after
Source: PLoS One. 2021 Sep 16;16(9):e0254240. doi: 10.1371/journal.pone.0254240 (PMC8445445; doi:10.1371/journal.pone.0254240)

In[290]:=
```
(* In order to make this notebook work it is necessary
 to first run the notebook "barbarigen n player". *)

(* This notebook shows outcomes for different combinations
 of parameter values r a c other than the best fit values
 used in barbarigen n player and barbarigen fit data. *)
```

In[291]:=
```
(* Here we use the best fit values again. *)
```

In[292]:=
```
r := .009;
a := .001;
c := 8;
tlag := 5;
```

In[296]:=
```
resourcehistory = FoldList[newresourcelist, initialresourcelist, Range[60]];
```

In[297]:=
```
ListPlot3D[resourcehistory, PlotRange → All, DataRange → {{0, 100}, {0, 1200}, {0, 6}},
 AxesLabel → {"distance", "year CE", "resources"},
 Ticks → {{0, 20, 40, 60, 80, 100}, {{0, "0"}, {200, ""}, {400, "400"},
    {600, ""}, {800, "800"}, {1000, ""}, {1200, "1200"}}, {2, 4, 6, 8, 10}},
 ColorFunction → GrayLevel, ColorFunctionScaling → {.4, 1}]
```

Out[297]=

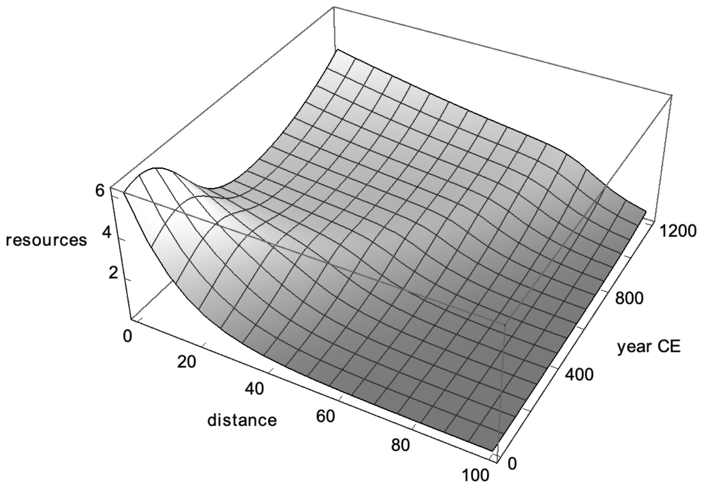

In[298]:=
```
(* Here we double the intrinsic rate of increase. *)
```

In[299]:=
```
r := .018;
a := .001;
c := 8;
tlag := 5;
```

In[303]:=
```
resourcehistory = FoldList[newresourcelist, initialresourcelist, Range[60]];
```

In[304]:=
```
ListPlot3D[resourcehistory, PlotRange → All, DataRange → {{0, 100}, {0, 1200}, {0, 6}},
 AxesLabel → {"distance", "year CE", "resources"},
 Ticks → {{0, 20, 40, 60, 80, 100}, {{0, "0"}, {200, ""}, {400, "400"},
    {600, ""}, {800, "800"}, {1000, ""}, {1200, "1200"}}, {2, 4, 6, 8, 10}},
 ColorFunction → GrayLevel, ColorFunctionScaling → {.4, 1}]
```

Out[304]=
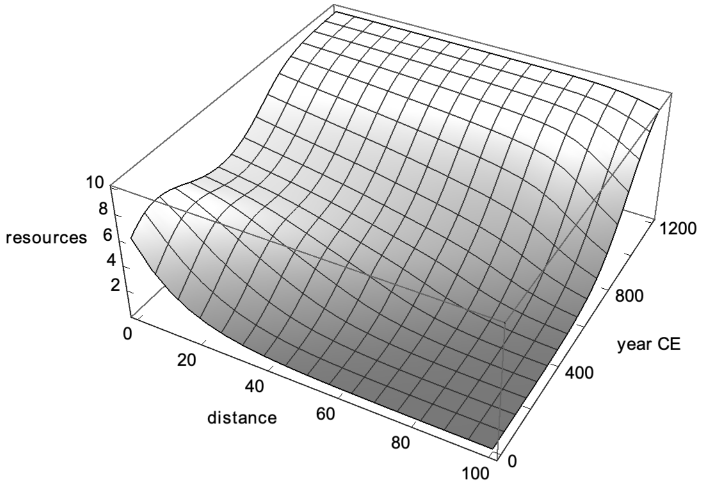

In[305]:=
```
(* Here we halve the intrinsic rate of increase. *)
```

In[306]:=
```
r := .0045;
a := .001;
c := 8;
tlag := 5;
```

In[310]:=
```
resourcehistory = FoldList[newresourcelist, initialresourcelist, Range[60]];
```

In[311]:=
```
ListPlot3D[resourcehistory, PlotRange → All, DataRange → {{0, 100}, {0, 1200}, {0, 6}},
 AxesLabel → {"distance", "year CE", "resources"},
 Ticks → {{0, 20, 40, 60, 80, 100}, {{0, "0"}, {200, ""}, {400, "400"},
    {600, ""}, {800, "800"}, {1000, ""}, {1200, "1200"}}, {2, 4, 6, 8, 10}},
 ColorFunction → GrayLevel, ColorFunctionScaling → {.4, 1}]
```

Out[311]=
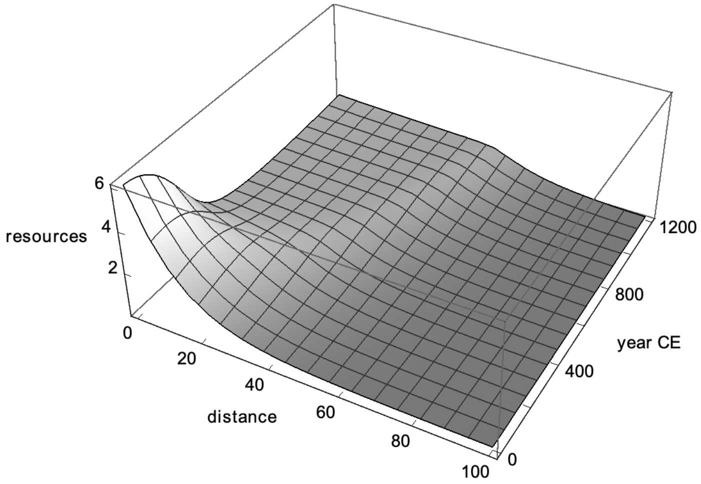

In[312]:=
```
(* Here we double the vulnerability to collateral damage. *)
```

In[313]:=
```
r := .009;
a := .001;
c := 16;
tlag := 5;
```

In[317]:=
```
resourcehistory = FoldList[newresourcelist, initialresourcelist, Range[60]];
```

In[318]:=
```
ListPlot3D[resourcehistory, PlotRange → All, DataRange → {{0, 100}, {0, 1200}, {0, 6}},
 AxesLabel → {"distance", "year CE", "resources"},
 Ticks → {{0, 20, 40, 60, 80, 100}, {{0, "0"}, {200, ""}, {400, "400"},
    {600, ""}, {800, "800"}, {1000, ""}, {1200, "1200"}}, {2, 4, 6, 8, 10}},
 ColorFunction → GrayLevel, ColorFunctionScaling → {.4, 1}]
```

Out[318]=

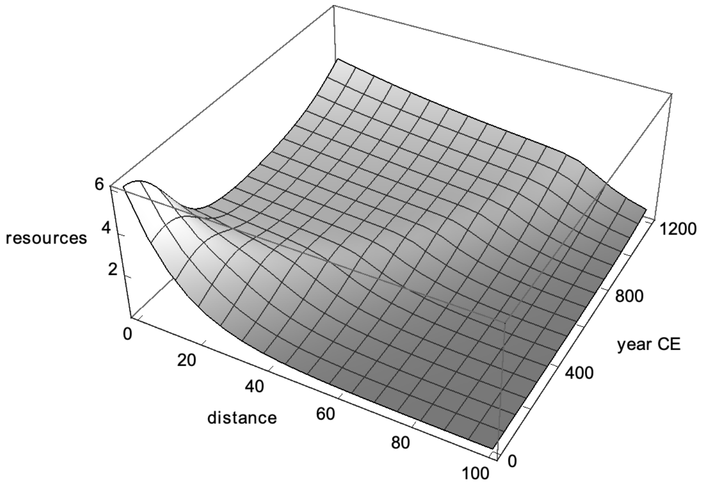

In[319]:=
```
(* Here we triple the vulnerability to collateral damage. *)
```

In[320]:=
```
r := .009;
a := .001;
c := 24;
tlag := 5;
```

In[324]:=
```
resourcehistory = FoldList[newresourcelist, initialresourcelist, Range[60]];
```

In[325]:=
```
ListPlot3D[resourcehistory, PlotRange → All, DataRange → {{0, 100}, {0, 1200}, {0, 6}},
 AxesLabel → {"distance", "year CE", "resources"},
 Ticks → {{0, 20, 40, 60, 80, 100}, {{0, "0"}, {200, ""}, {400, "400"},
    {600, ""}, {800, "800"}, {1000, ""}, {1200, "1200"}}, {2, 4, 6, 8, 10}},
 ColorFunction → GrayLevel, ColorFunctionScaling → {.4, 1}]
```

Out[325]=

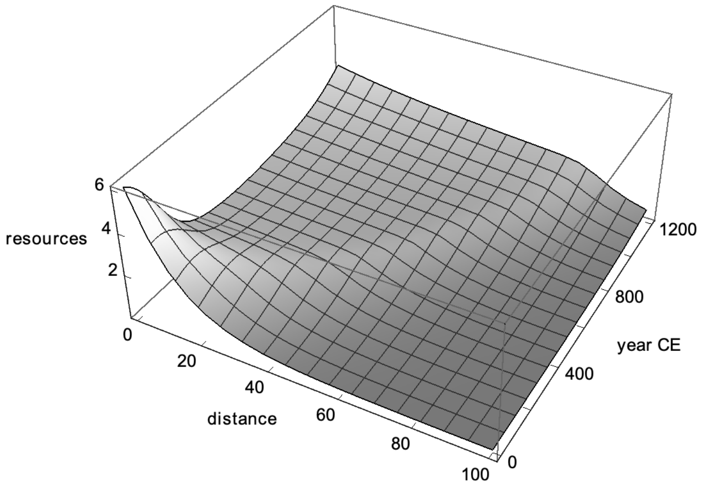

In[326]:=
```
(* Here we halve the vulnerability to collateral damage. *)
```

In[327]:=
```
r := .009;
a := .001;
c := 4;
tlag := 5;
```

In[331]:=
```
resourcehistory = FoldList[newresourcelist, initialresourcelist, Range[60]];
```

In[332]:=
```
ListPlot3D[resourcehistory, PlotRange → All, DataRange → {{0, 100}, {0, 1200}, {0, 6}},
 AxesLabel → {"distance", "year CE", "resources"},
 Ticks → {{0, 20, 40, 60, 80, 100}, {{0, "0"}, {200, ""}, {400, "400"},
    {600, ""}, {800, "800"}, {1000, ""}, {1200, "1200"}}, {2, 4, 6, 8, 10}},
 ColorFunction → GrayLevel, ColorFunctionScaling → {.4, 1}]
```

Out[332]=

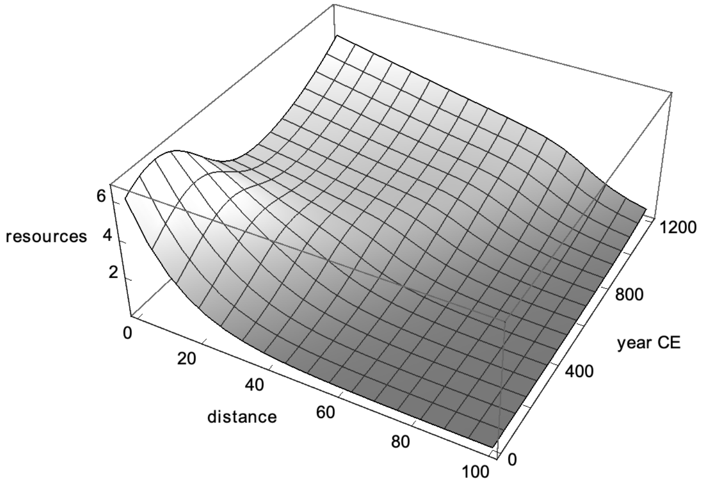

In[333]:=
```
(* Here we eliminate vulnerability to collateral damage altogether. *)
```

In[334]:=
```
r := .009;
a := .001;
c := 0;
tlag := 5;
```

In[338]:=
```
resourcehistory = FoldList[newresourcelist, initialresourcelist, Range[60]];
```

In[339]:=
```
ListPlot3D[resourcehistory, PlotRange → All, DataRange → {{0, 100}, {0, 1200}, {0, 6}},
 AxesLabel → {"distance", "year CE", "resources"},
 Ticks → {{0, 20, 40, 60, 80, 100}, {{0, "0"}, {200, ""}, {400, "400"},
    {600, ""}, {800, "800"}, {1000, ""}, {1200, "1200"}}, {2, 4, 6, 8, 10}},
 ColorFunction → GrayLevel, ColorFunctionScaling → {.4, 1}]
```

Out[339]=
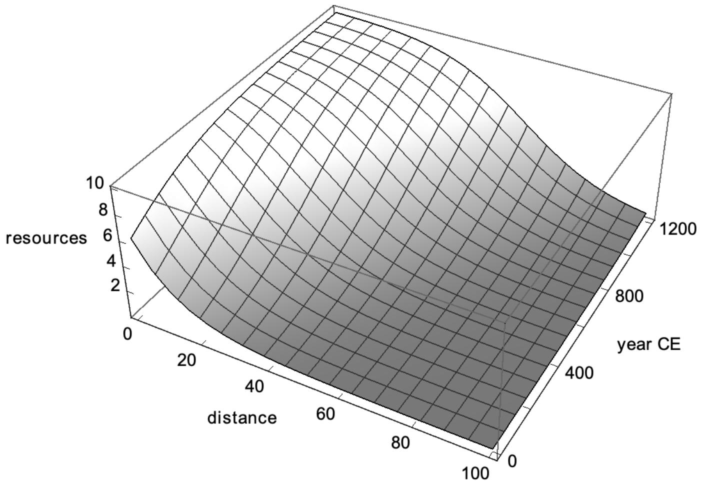

In[340]:=
```
(* Here we greatly increase the rate of diffusion. *)
```

In[341]:=
```
r := .009;
a := .1;
c := 8;
tlag := 5;
```

In[345]:=
```
resourcehistory = FoldList[newresourcelist, initialresourcelist, Range[60]];
```

In[346]:=
```
ListPlot3D[resourcehistory, PlotRange → All, DataRange → {{0, 100}, {0, 1200}, {0, 6}},
 AxesLabel → {"distance", "year CE", "resources"},
 Ticks → {{0, 20, 40, 60, 80, 100}, {{0, "0"}, {200, ""}, {400, "400"},
    {600, ""}, {800, "800"}, {1000, ""}, {1200, "1200"}}, {2, 4, 6, 8, 10}},
 ColorFunction → GrayLevel, ColorFunctionScaling → {.4, 1}]
```

Out[346]=

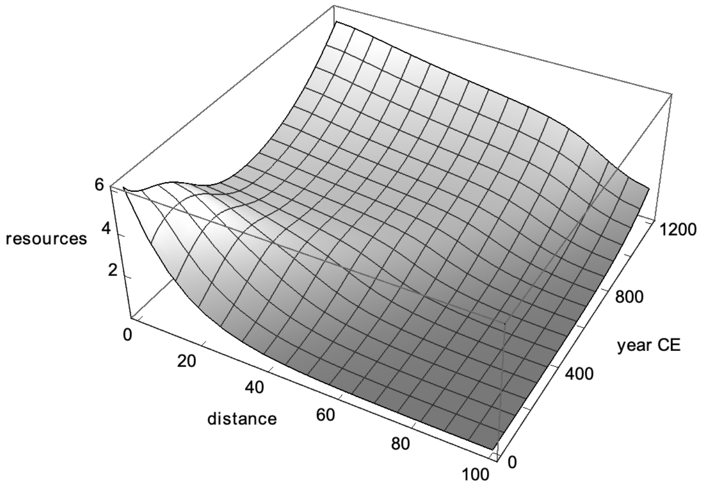

Supplement: S3 File — (PDF) [file pone.0254240.s003.pdf]
