# Supplementary material for: Barbarigenesis and the collapse of complex societies: Rome and after
Source: PLoS One. 2021 Sep 16;16(9):e0254240. doi: 10.1371/journal.pone.0254240 (PMC8445445; doi:10.1371/journal.pone.0254240)

```
In[35]:= (* This notebook shows outcomes for the 3 person game given in
          Equation xx and the spatial game. It is the basis Figures 2 thru 4. *)

In[36]:= (* OUTLINE
          PART ONE. 3 players with resources 10, and 4 and 3
          PART TWO. 3 players with resources x and y and z. Figure 2a and 2b
          PART THREE. A game in space. Figure 3
          PART FOUR. A game in space and time. Figures 4a and 4b
         *)

In[37]:= (* PART ONE. 3 players with resources 10, 4 and 3 *)

In[38]:= Clear[r, n, def, f, fd, ft, opt, optlist, constraintlist1,
          constraintlist2, constraintlist3, constraintlist4, flist,
          λlist, σlist, λijlist, σijlist, initiallist, result]

In[39]:= (* We consider the case where n=3 *)

In[40]:= r := {10, 4, 3}

In[41]:= n := Length[r]

In[42]:= (* Variables are
          r is a vector giving the resources of n players
          r[[j]] is the resources of player j.
           f[i,j] is the fighting effort of player i against player j.
           fd[j] is the total fighting effort player i directs against everybody
          ft[j] is the total fighting effort everybody directs against player j.
           Note that here the exponent m,
         the decisiveness of conflict, is set to 1 and omitted *)

In[43]:= fd[i_] := Total[Drop[Table[f[i, j], {j, n}], {i}]]

In[44]:= ft[i_] := Total[Drop[Table[f[j, i], {j, n}], {i}]]

In[45]:= (* First we set various variables and function values to 0,
         as appropriate. You don't fight yourself - f[i,i]=0 - etc. *)

In[46]:= opt[i_, i_, rr_] := 0;
         f[i_, i_] := 0;
         σ[i_, i_] := 0;
         f[i_, n + 1] := 0;
         λ[i_, i_] := 0
```

```
In[47]:= (* Each player i tries to maximize his income. We want
          to figure out the optimum f[i,j] for all players i and j.
           The income that i gets from j is
          f[i,j]/(fd[j]+ft[j]) (r[[j]]-fd[j])
          The portion of player i's own production that he hangs onto is
          fd[i]/(fd[i]+ft[i]) (r[[i]]-fd[i]).
           When we add these two income sources and take
          the derivative with respect to f[i,j], given the vector r,
         and tack on some Lagrange multipliers (see constraintlist below),
         we get the function opt[i,j,r] which we will be setting to 0.
          *)
```

In[48]:= $$\text{opt[i\_, j\_, rr\_] :=}\ \frac{\text{rr[[j]] - fd[j]}}{\text{fd[j] + ft[j]}} - \text{f[i, j]}\ \frac{\text{rr[[j]] - fd[j]}}{(\text{fd[j] + ft[j]})^2} + \frac{\text{rr[[i]] - fd[i]}}{\text{fd[i] + ft[i]}} - \text{fd[i]}\ \frac{\text{rr[[i]] - fd[i]}}{(\text{fd[i] + ft[i]})^2} - \text{fd[i]}\ \frac{1}{\text{fd[i] + ft[i]}} + \lambda\text{[i]} + \lambda\text{[i, j]}$$

```
In[49]:= (* We can make a list of opt[i,j,r] for all i and j *)

In[50]:= optlist[rr_] := Flatten[Table[Drop[Table[opt[i, j, rr], {j, n}], {i}], {i, n}]]

In[51]:= (* Maximization is subject to constraints
                 f[i,j]-σ[i,j]^2>=0 (fighting effort is nonnegative)
               r[i]]-fd[i]-σ[i]^2>=0 (production is nonnegative)
            We list various constraints below
            to handle corner solutions via Lagrange multipliers.)
          *)

In[52]:= constraintlist1[rr_] := Table[rr[[i]] - fd[i] - σ[i]^2, {i, n}]

In[53]:= constraintlist2 := Table[2 σ[i] λ[i], {i, n}]

In[54]:= constraintlist3 := Flatten[Table[Drop[Table[f[i, j] - σ[i, j]^2, {j, n}], {i}], {i, n}]]

In[55]:= constraintlist4 := Flatten[Table[Drop[Table[2 σ[i, j] λ[i, j], {j, n}], {i}], {i, n}]]

In[56]:= flist := Flatten[Table[Drop[Table[f[i, j], {j, n}], {i}], {i, n}]]

In[57]:= λlist := Table[λ[i], {i, n}]

In[58]:= σlist := Table[σ[i], {i, n}]

In[59]:= λijlist := Flatten[Table[Drop[Table[λ[i, j], {j, n}], {i}], {i, n}]]

In[60]:= σijlist := Flatten[Table[Drop[Table[σ[i, j], {j, n}], {i}], {i, n}]]

In[61]:= (* We need to give Mathematica's FindRoot
          function some initial values to work with *)
```

```
In[62]:= initiallist1 := Join[Transpose[{flist, ConstantArray[1, n^2 - n]}],
           Transpose[{λlist, ConstantArray[-.01, n]}],
           Transpose[{σlist, ConstantArray[.1, n]}],
           Transpose[{λijlist, ConstantArray[.1, n^2 - n]}],
           Transpose[{σijlist, ConstantArray[.1, n^2 - n]}]]

In[63]:= (* We define the function result[r,initial] which gives the equlibrium income
          for each player. We find the solution starting with the initial values. *)

In[64]:= result[rr_, initiall_] := FindRoot[Join[optlist[rr], constraintlist1[rr],
            constraintlist2, constraintlist3, constraintlist4], initiall]

In[65]:= (* Here are results for r={10,4,3} *)

In[66]:= result[r, initiallist1]
```

Out[66]= $\{f[1,2] \to 2.50576,\ f[1,3] \to 1.70859,\ f[2,1] \to 2.8517,\ f[2,3] \to -1.85212\times10^{-20},$
$f[3,1] \to 2.20742,\ f[3,2] \to -1.11803\times10^{-23},\ \lambda[1] \to 1.85991\times10^{-33},$
$\lambda[2] \to -1.84753\times10^{-29},\ \lambda[3] \to -8.91906\times10^{-28},\ \sigma[1] \to 2.40534,\ \sigma[2] \to 1.07159,$
$\sigma[3] \to 0.89027,\ \lambda[1,2] \to 1.51277\times10^{-30},\ \lambda[1,3] \to 8.64926\times10^{-30},$
$\lambda[2,1] \to 4.54423\times10^{-31},\ \lambda[2,3] \to 0.229644,\ \lambda[3,1] \to -5.80835\times10^{-34},$
$\lambda[3,2] \to 0.261048,\ \sigma[1,2] \to 1.58296,\ \sigma[1,3] \to 1.30713,\ \sigma[2,1] \to 1.6887,$
$\sigma[2,3] \to 1.49621\times10^{-20},\ \sigma[3,1] \to 1.48574,\ \sigma[3,2] \to -1.13839\times10^{-22}\}$

```
In[67]:= (* To make the output readable we define two functions -
          fmatrix[r,initiallist] and payoff[r,initiallist] - that give the fighting
           effort for each pair of players and the income for each player *)

In[68]:= fmatrix[rr_, initiall_] := Table[f[i, j], {i, 1, n}, {j, 1, n}] /. result[rr, initiall]

In[69]:= payoff[rr_, initiall_] :=
```

$$\text{Table}\Big[\text{Total}\Big[\text{Drop}\Big[\text{Table}\Big[f[i,j]\,\frac{rr[[j]]-fd[j]}{fd[j]+ft[j]},\ \{j,1,n\}\Big],\ \{i\}\Big]\Big]+ fd[i]\,\frac{rr[[i]]-fd[i]}{fd[i]+ft[i]},\ \{i,1,n\}\Big]\ /.\ \text{result}[rr,\ initiall]$$

```
In[70]:= (* Here's what they give us for r={10,4,3}) *)

In[71]:= MatrixForm[fmatrix[r, initiallist1]]
```

Out[71]//MatrixForm=

$$\begin{pmatrix} 0 & 2.50576 & 1.70859 \\ 2.8517 & 0 & -1.85212\times10^{-20} \\ 2.20742 & -1.11803\times10^{-23} & 0 \end{pmatrix}$$

```
In[72]:= payoff[r, initiallist1]

Out[72]= {3.51219, 2.39038, 1.82396}

In[73]:= (* PART TWO. 3 players with resources x and y and z  *)
```

```
In[74]:= (* Above we calculated results for a single case r=
            {10,4,3}. Below we calculate general results for r=(10,y,z}. We
         proceed in multiple steps. We have to use a different set of initial
         values in some cases to get Mathematica's FindRoot runction to
         behave itself. We have to do a some special work to get values near
         and at the edges. Then we turn a half table into a symmetrical full
         table. Finally we use interpolation to get a continuous function. *)

In[75]:= (* First, we come up with a new list of initial conditions - initiallist 2 -
         to make FindRoot behave itself in the neighborhood of some corner solutions *)

In[76]:= seedinitial2 := result[{10, 4, 3}, initiallist1]

In[77]:= initiallist2 := Transpose[{Join[flist, λlist, σlist, λijlist, σijlist],
           Join[flist, λlist, σlist, λijlist, σijlist] /. seedinitial2}]

In[78]:= (* We use our different initial values as appropriate to construct two triangular
         tables giving payoffs and fighting effort where the resources of the first
         player are always 10 and the resources of the second player (index i) go
         from 10 to 1 and the resources of the third player (index j) go from 10
         to i. In halftablepayoff12 each column of three numbers is the payoff to
         players 1 2 and 3 for a given combination of resources 10 and i and j *)

In[79]:= halftablepayoff12 =
          Join[Table[payoff[{10, i, j}, initiallist1], {i, 10, 4, -1}, {j, i, 1, -1}],
           Table[payoff[{10, i, j}, initiallist2], {i, 3, 3, -1}, {j, i, 1, -1}],
           Table[payoff[{10, i, j}, initiallist1], {i, 2, 1, -1}, {j, i, 1, -1}]];
```

In[80]:=
```
TableForm[%]
```

Out[80]//TableForm=

4.44444<br>4.44444<br>4.44444	4.4294<br>4.4294<br>4.03822	4.42499<br>4.42499<br>3.6279	4.43235<br>4.43235<br>3.21264	4.45291<br>4.45291<br>2.79134	4.48839<br>4.48839<br>2.36257	4.54102<br>4.54102<br>1.92437	4.61[illegible]<br>4.61[illegible]<br>1.47[illegible]
4.40263<br>4.0252<br>4.0252	4.38607<br>4.02333<br>3.61707	4.38088<br>4.03382<br>3.2042	4.38847<br>4.05814<br>2.78552	4.41058<br>4.09811<br>2.3596	4.44941<br>4.15604<br>1.92453	4.50785<br>4.23496<br>1.47762	4.58[illegible]<br>4.33[illegible]<br>1.01[illegible]
4.3569<br>3.61802<br>3.61802	4.33862<br>3.632<br>3.20829	4.33262<br>3.66054<br>2.79314	4.34061<br>3.70552<br>2.37127	4.36476<br>3.76932<br>1.94092	4.40782<br>3.855<br>1.49969	4.47219<br>3.96702<br>1.04444	4.45[illegible]<br>4.17[illegible]<br>0.56[illegible]
4.3067<br>3.22622<br>3.22622	4.28647<br>3.25956<br>2.81561	4.2796<br>3.31027<br>2.39907	4.28818<br>3.38072<br>1.97519	4.31477<br>3.4739<br>1.54215	4.27491<br>3.63113<br>1.10183	4.18475<br>3.88222<br>0.606472	
4.25134<br>2.85456<br>2.85456	4.22888<br>2.91198<br>2.44474	4.22105<br>2.99022<br>2.02923	4.14206<br>3.11355<br>1.61353	3.96139<br>3.30877<br>1.16969	3.88582<br>3.58336<br>0.652603		
4.19006<br>2.51032<br>2.51032	4.07593<br>2.61055<br>2.11341	3.82714<br>2.76394<br>1.70744	3.63883<br>2.97063<br>1.25195	3.57782<br>3.27425<br>0.708503			
3.78223<br>2.23442<br>2.23442	3.51219<br>2.39038<br>1.82396	3.30627<br>2.60996<br>1.3547	3.25884<br>2.95241<br>0.776708				
3.20928<br>1.97825<br>1.97825	2.96444<br>2.21152<br>1.49185	3.03337<br>2.61249<br>0.870829					
3.03337<br>1.74166<br>1.74166	3.51<br>2.16333<br>1.08167						
4.20204<br>1.44949<br>1.44949							

In[81]:=
```
(* In halftablefight12 each column of three numbers is the fighting effort of
 players 1 2 and 3 for a given combination of resources 10 and i and j *)
```

In[82]:=
```
halftablefight12 = Join[
   Table[fmatrix[{10, i, j}, initiallist1].{1, 1, 1}, {i, 10, 4, -1}, {j, i, 1, -1}],
   Table[fmatrix[{10, i, j}, initiallist2].{1, 1, 1}, {i, 3, 3, -1}, {j, i, 1, -1}],
   Table[fmatrix[{10, i, j}, initiallist1].{1, 1, 1}, {i, 2, 1, -1}, {j, i, 1, -1}]];
```

In[83]:=
```
TableForm[%]
```

Out[83]//TableForm=

5.55556<br>5.55556<br>5.55556	5.5137<br>5.5137<br>5.07558	5.46994<br>5.46994<br>4.58224	5.42404<br>5.42404<br>4.07458	5.37569<br>5.37569<br>3.55147	5.3245<br>5.3245<br>3.01164	5.26997<br>5.26997<br>2.45363	5.211[illegible]<br>5.211[illegible]<br>1.875[illegible]
5.46683<br>5.04007<br>5.04007	5.41753<br>5.00318<br>4.55282	5.36539<br>4.96478<br>4.05091	5.30995<br>4.92474<br>3.53318	5.25056<br>4.88289<br>2.99827	5.18633<br>4.83908<br>2.44461	5.11603<br>4.79316<br>1.87039	5.037[illegible]<br>4.745[illegible]<br>1.273[illegible]
5.36206<br>4.5225<br>4.5225	5.30302<br>4.49127<br>4.02679	5.23973<br>4.45915<br>3.51481	5.17124<br>4.42624<br>2.98511	5.09626<br>4.39273<br>2.43601	5.0129<br>4.35906<br>1.86553	4.91746<br>4.32728<br>1.27161	4.726[illegible]<br>4.407[illegible]<br>0.666[illegible]
5.23625<br>4.00231<br>4.00231	5.16414<br>3.97766<br>3.49656	5.08545<br>3.95315<br>2.97246	4.99839<br>3.92937<br>2.42815	4.90033<br>3.90737<br>1.86149	4.73297<br>3.97446<br>1.28469	4.48869<br>4.13478<br>0.703085	
5.082<br>3.47877<br>3.47877	4.99167<br>3.46197<br>2.96075	4.8908<br>3.44712<br>2.42157	4.72708<br>3.51335<br>1.89043	4.47846<br>3.69927<br>1.38241	4.23597<br>3.88051<br>0.761739		
4.88781<br>2.95075<br>2.95075	4.72396<br>3.00933<br>2.46682	4.47137<br>3.19892<br>2.03119	4.22258<br>3.41323<br>1.50277	3.98275<br>3.6251<br>0.831577			
4.46884<br>2.64004<br>2.64004	4.21435<br>2.8517<br>2.20742	3.96466<br>3.10721<br>1.65721	3.72911<br>3.36779<br>0.915139				
3.95651<br>2.43885<br>2.43885	3.70337<br>2.75613<br>1.8727	3.48331<br>3.<br>1.					
3.48331<br>2.<br>2.	3.245<br>2.<br>1.						
2.89898<br>1.<br>1.							

In[84]:=
```
(* Again we come up with a new list of initial conditions -
 initiallist3 - to make FindRoot behave itself near the
  edge where resources for the weakest player equal .5 *)
```

In[85]:=
```
Clear[seedinitial3]
```

In[86]:=
```
seedinitial3 = Reverse[Join[Table[result[{10, i, 1}, initiallist1], {i, 10, 4, -1}],
    Table[result[{10, i, 1}, initiallist1], {i, 3, 3, -1}],
    Table[result[{10, i, 1}, initiallist1], {i, 2, 1, -1}]]];
```

In[87]:=
```
initiallist3[i_] := Transpose[{Join[flist, λlist, σlist, λijlist, σijlist],
   Join[flist, λlist, σlist, λijlist, σijlist] /. seedinitial3[[i]]}]
```

In[88]:=
```
(* We construct two tables - halftablepayoff3 and halftablefight3 -
 for contests near the edge where resources for the weakest player equal .5 *)
```

In[89]:=
```
halftablepayoff3 = Table[payoff[{10, i, .5}, initiallist3[i]], {i, 10, 1, -1}];
```

In[90]:=
```
halftablefight3 =
  Table[fmatrix[{10, i, .5}, initiallist3[i]].{1, 1, 1}, {i, 10, 1, -1}];
```

In[91]:=
```
(* Then we join our various tables to get two bigger tables
 that cover everything from 10 to .5 for the weaker players *)
```

In[92]:=
```
halftablepayoff123 =
  Append[MapThread[Append, {halftablepayoff12, halftablepayoff3}],
   {payoff[{10, .5, .5}, initiallist1]}];
```

In[93]:=
```
% // TableForm
```

Out[93]//TableForm=

4.44444<br>4.44444<br>4.44444	4.4294<br>4.4294<br>4.03822	4.42499<br>4.42499<br>3.6279	4.43235<br>4.43235<br>3.21264	4.45291<br>4.45291<br>2.79134	4.48839<br>4.48839<br>2.36257	4.54102<br>4.54102<br>1.92437	4.6[illegible]<br>4.6[illegible]<br>1.4[illegible]
4.40263<br>4.0252<br>4.0252	4.38607<br>4.02333<br>3.61707	4.38088<br>4.03382<br>3.2042	4.38847<br>4.05814<br>2.78552	4.41058<br>4.09811<br>2.3596	4.44941<br>4.15604<br>1.92453	4.50785<br>4.23496<br>1.47762	4.5[illegible]<br>4.3[illegible]<br>1.0[illegible]
4.3569<br>3.61802<br>3.61802	4.33862<br>3.632<br>3.20829	4.33262<br>3.66054<br>2.79314	4.34061<br>3.70552<br>2.37127	4.36476<br>3.76932<br>1.94092	4.40782<br>3.855<br>1.49969	4.47219<br>3.96702<br>1.04444	4.4[illegible]<br>4.1[illegible]<br>0.5[illegible]
4.3067<br>3.22622<br>3.22622	4.28647<br>3.25956<br>2.81561	4.2796<br>3.31027<br>2.39907	4.28818<br>3.38072<br>1.97519	4.31477<br>3.4739<br>1.54215	4.27491<br>3.63113<br>1.10183	4.18475<br>3.88222<br>0.606472	4.1[illegible]<br>4.0[illegible]<br>0.3[illegible]
4.25134<br>2.85456<br>2.85456	4.22888<br>2.91198<br>2.44474	4.22105<br>2.99022<br>2.02923	4.14206<br>3.11355<br>1.61353	3.96139<br>3.30877<br>1.16969	3.88582<br>3.58336<br>0.652603	3.91246<br>3.76843<br>0.349191	
4.19006<br>2.51032<br>2.51032	4.07593<br>2.61055<br>2.11341	3.82714<br>2.76394<br>1.70744	3.63883<br>2.97063<br>1.25195	3.57782<br>3.27425<br>0.708503	3.62714<br>3.48446<br>0.382173		
3.78223<br>2.23442<br>2.23442	3.51219<br>2.39038<br>1.82396	3.30627<br>2.60996<br>1.3547	3.25884<br>2.95241<br>0.776708	3.33576<br>3.19468<br>0.421651			
3.20928<br>1.97825<br>1.97825	2.96444<br>2.21152<br>1.49185	3.03337<br>2.61249<br>0.870829	3.34649<br>3.01399<br>0.496943				
3.03337<br>1.74166<br>1.74166	3.51<br>2.16333<br>1.08167	3.81966<br>2.47214<br>0.618034					
4.20204<br>1.44949<br>1.44949	4.69338<br>1.76887<br>0.884437						
5.36675<br>1.15831<br>1.15831							

In[94]:=
```
halftablefight123 = Append[MapThread[Append, {halftablefight12, halftablefight3}],
   {fmatrix[{10, .5, .5}, initiallist1].{1, 1, 1}}];
```

In[95]:=
```
% // TableForm
```

Out[95]//TableForm=

5.55556	5.5137	5.46994	5.42404	5.37569	5.3245	5.26997	5.2114
5.55556	5.5137	5.46994	5.42404	5.37569	5.3245	5.26997	5.2114
5.55556	5.07558	4.58224	4.07458	3.55147	3.01164	2.45363	1.8757
5.46683	5.41753	5.36539	5.30995	5.25056	5.18633	5.11603	5.0377
5.04007	5.00318	4.96478	4.92474	4.88289	4.83908	4.79316	4.7450
5.04007	4.55282	4.05091	3.53318	2.99827	2.44461	1.87039	1.2734
5.36206	5.30302	5.23973	5.17124	5.09626	5.0129	4.91746	4.7268
4.5225	4.49127	4.45915	4.42624	4.39273	4.35906	4.32728	4.407[illegible]
4.5225	4.02679	3.51481	2.98511	2.43601	1.86553	1.27161	0.666[illegible]
5.23625	5.16414	5.08545	4.99839	4.90033	4.73297	4.48869	4.3687
4.00231	3.97766	3.95315	3.92937	3.90737	3.97446	4.13478	4.2020
4.00231	3.49656	2.97246	2.42815	1.86149	1.28469	0.703085	0.3680
5.082	4.99167	4.8908	4.72708	4.47846	4.23597	4.11762	
3.47877	3.46197	3.44712	3.51335	3.69927	3.88051	3.95351	
3.47877	2.96075	2.42157	1.89043	1.38241	0.761739	0.398782	
4.88781	4.72396	4.47137	4.22258	3.98275	3.86664		
2.95075	3.00933	3.19892	3.41323	3.6251	3.706		
2.95075	2.46682	2.03119	1.50277	0.831577	0.433592		
4.46884	4.21435	3.96466	3.72911	3.61634			
2.64004	2.8517	3.10721	3.36779	3.45984			
2.64004	2.20742	1.65721	0.915139	0.471733			
3.95651	3.70337	3.48331	3.32676				
2.43885	2.75613	3.	3.				
2.43885	1.8727	1.	0.315827				
3.48331	3.245	3.09017					
2.	2.	2.					
2.	1.	0.5					
2.89898	2.65331						
1.	1.						
1.	0.5						
2.31662							
0.5							
0.5							

In[96]:=
```
(* We construct two more tables - halftablepayoff4 and halftablefight4 -
   for contests right at the edge where resources for the weakest
    player equal 0. This amounts to a 2 player game so we set n=2*)
```

In[97]:=
```
n := 2
```

In[98]:=
```
halftablepayoff4 =
  Append[Table[Append[payoff[{10, i}, initiallist1], 0], {i, 10, 1, -1}],
   Append[payoff[{10, .5, 0}, initiallist1], 0]]
```

Out[98]=
```
{{5., 5., 0}, {4.75, 4.75, 0}, {4.5, 4.5, 0}, {4.25, 4.25, 0},
 {4., 4., 0}, {3.75, 3.75, 0}, {3.5, 3.5, 0}, {3.51, 3.245, 0},
 {4.20204, 2.89898, 0}, {5.36675, 2.31662, 0}, {6.41742, 1.79129, 0}}
```

In[99]:=
```
halftablefight4 = Map[Append[#, 0] &,
   Append[Table[Map[Total, fmatrix[{10, i}, initiallist1]], {i, 10, 1, -1}],
    Map[Total, fmatrix[{10, .5, 0}, initiallist1]]]]
```

Out[99]=
```
{{5., 5., 0}, {4.75, 4.75, 0}, {4.5, 4.5, 0},
 {4.25, 4.25, 0}, {4., 4., 0}, {3.75, 3.75, 0}, {3.5, 3.5, 0},
 {3.245, 3., 0}, {2.89898, 2., 0}, {2.31662, 1., 0}, {1.79129, 0.5, 0}}
```

In[100]:=
```
(* Then we join our various tables to get two even bigger
 tables that cover everything from 10 to 0 for the weaker players*)
```

In[101]:=
```
halftablepayoff1234 =
  Append[MapThread[Append, {halftablepayoff123, halftablepayoff4}], {{10, 0, 0}}] //
   TableForm
```

Out[101]//TableForm=

4.44444<br>4.44444<br>4.44444	4.4294<br>4.4294<br>4.03822	4.42499<br>4.42499<br>3.6279	4.43235<br>4.43235<br>3.21264	4.45291<br>4.45291<br>2.79134	4.48839<br>4.48839<br>2.36257	4.54102<br>4.54102<br>1.92437	4.6[illegible]<br>4.6[illegible]<br>1.47[illegible]
4.40263<br>4.0252<br>4.0252	4.38607<br>4.02333<br>3.61707	4.38088<br>4.03382<br>3.2042	4.38847<br>4.05814<br>2.78552	4.41058<br>4.09811<br>2.3596	4.44941<br>4.15604<br>1.92453	4.50785<br>4.23496<br>1.47762	4.58[illegible]<br>4.33[illegible]<br>1.01[illegible]
4.3569<br>3.61802<br>3.61802	4.33862<br>3.632<br>3.20829	4.33262<br>3.66054<br>2.79314	4.34061<br>3.70552<br>2.37127	4.36476<br>3.76932<br>1.94092	4.40782<br>3.855<br>1.49969	4.47219<br>3.96702<br>1.04444	4.45[illegible]<br>4.17[illegible]<br>0.56[illegible]
4.3067<br>3.22622<br>3.22622	4.28647<br>3.25956<br>2.81561	4.2796<br>3.31027<br>2.39907	4.28818<br>3.38072<br>1.97519	4.31477<br>3.4739<br>1.54215	4.27491<br>3.63113<br>1.10183	4.18475<br>3.88222<br>0.606472	4.19[illegible]<br>4.04[illegible]<br>0.32[illegible]
4.25134<br>2.85456<br>2.85456	4.22888<br>2.91198<br>2.44474	4.22105<br>2.99022<br>2.02923	4.14206<br>3.11355<br>1.61353	3.96139<br>3.30877<br>1.16969	3.88582<br>3.58336<br>0.652603	3.91246<br>3.76843<br>0.349191	4.<br>4.<br>0
4.19006<br>2.51032<br>2.51032	4.07593<br>2.61055<br>2.11341	3.82714<br>2.76394<br>1.70744	3.63883<br>2.97063<br>1.25195	3.57782<br>3.27425<br>0.708503	3.62714<br>3.48446<br>0.382173	3.75<br>3.75<br>0	
3.78223<br>2.23442<br>2.23442	3.51219<br>2.39038<br>1.82396	3.30627<br>2.60996<br>1.3547	3.25884<br>2.95241<br>0.776708	3.33576<br>3.19468<br>0.421651	3.5<br>3.5<br>0		
3.20928<br>1.97825<br>1.97825	2.96444<br>2.21152<br>1.49185	3.03337<br>2.61249<br>0.870829	3.34649<br>3.01399<br>0.496943	3.51<br>3.245<br>0			
3.03337<br>1.74166<br>1.74166	3.51<br>2.16333<br>1.08167	3.81966<br>2.47214<br>0.618034	4.20204<br>2.89898<br>0				
4.20204<br>1.44949<br>1.44949	4.69338<br>1.76887<br>0.884437	5.36675<br>2.31662<br>0					
5.36675<br>1.15831<br>1.15831	6.41742<br>1.79129<br>0						
10<br>0<br>0							

In[102]:=
```
halftablefight1234 = Append[
   MapThread[Append, {halftablefight123, halftablefight4}], {{0, 0, 0}}] // TableForm
```

Out[102]//TableForm=

5.55556	5.5137	5.46994	5.42404	5.37569	5.3245	5.26997	5.211
5.55556	5.5137	5.46994	5.42404	5.37569	5.3245	5.26997	5.211
5.55556	5.07558	4.58224	4.07458	3.55147	3.01164	2.45363	1.875
5.46683	5.41753	5.36539	5.30995	5.25056	5.18633	5.11603	5.037
5.04007	5.00318	4.96478	4.92474	4.88289	4.83908	4.79316	4.745
5.04007	4.55282	4.05091	3.53318	2.99827	2.44461	1.87039	1.273
5.36206	5.30302	5.23973	5.17124	5.09626	5.0129	4.91746	4.726
4.5225	4.49127	4.45915	4.42624	4.39273	4.35906	4.32728	4.407
4.5225	4.02679	3.51481	2.98511	2.43601	1.86553	1.27161	0.666
5.23625	5.16414	5.08545	4.99839	4.90033	4.73297	4.48869	4.368
4.00231	3.97766	3.95315	3.92937	3.90737	3.97446	4.13478	4.202
4.00231	3.49656	2.97246	2.42815	1.86149	1.28469	0.703085	0.368
5.082	4.99167	4.8908	4.72708	4.47846	4.23597	4.11762	4.
3.47877	3.46197	3.44712	3.51335	3.69927	3.88051	3.95351	4.
3.47877	2.96075	2.42157	1.89043	1.38241	0.761739	0.398782	0
4.88781	4.72396	4.47137	4.22258	3.98275	3.86664	3.75	
2.95075	3.00933	3.19892	3.41323	3.6251	3.706	3.75	
2.95075	2.46682	2.03119	1.50277	0.831577	0.433592	0	
4.46884	4.21435	3.96466	3.72911	3.61634	3.5		
2.64004	2.8517	3.10721	3.36779	3.45984	3.5		
2.64004	2.20742	1.65721	0.915139	0.471733	0		
3.95651	3.70337	3.48331	3.32676	3.245			
2.43885	2.75613	3.	3.	3.			
2.43885	1.8727	1.	0.315827	0			
3.48331	3.245	3.09017	2.89898				
2.	2.	2.	2.				
2.	1.	0.5	0				
2.89898	2.65331	2.31662					
1.	1.	1.					
1.	0.5	0					
2.31662	1.79129						
0.5	0.5						
0.5	0						
0							
0							
0							

In[103]:=
```
(* Because the situation of the two weaker players is symmetrical
  we can create some functions - switchtrio and tableentry -
 that let us turn our triangular tables into square tables. *)
```

In[104]:=
```
Clear[switchtrio]
```

In[105]:=
```
switchtrio[list_] := {list[[1]], list[[3]], list[[2]]}
```

In[106]:=
```
Clear[tableentry]
```

In[107]:=
```
tableentry[i_, j_, list_] := list[[1, i, j - i + 1]] /; i <= j
```

In[108]:=
```
tableentry[i_, j_, list_] := switchtrio[list[[1, j, -j + i + 1]]] /; i > j
```

In[109]:=
```
fulltablepayoff := Table[tableentry[i, j, halftablepayoff1234], {i, 12}, {j, 12}];
```

In[110]:= fulltablepayoff // TableForm

Out[110]//TableForm=

4.44444	4.4294	4.42499	4.43235	4.45291	4.48839	4.54102	4.[illegible]
4.44444	4.4294	4.42499	4.43235	4.45291	4.48839	4.54102	4.[illegible]
4.44444	4.03822	3.6279	3.21264	2.79134	2.36257	1.92437	1.[illegible]
4.4294	4.40263	4.38607	4.38088	4.38847	4.41058	4.44941	4.[illegible]
4.03822	4.0252	4.02333	4.03382	4.05814	4.09811	4.15604	4.[illegible]
4.4294	4.0252	3.61707	3.2042	2.78552	2.3596	1.92453	1.[illegible]
4.42499	4.38607	4.3569	4.33862	4.33262	4.34061	4.36476	4.[illegible]
3.6279	3.61707	3.61802	3.632	3.66054	3.70552	3.76932	3.[illegible]
4.42499	4.02333	3.61802	3.20829	2.79314	2.37127	1.94092	1.[illegible]
4.43235	4.38088	4.33862	4.3067	4.28647	4.2796	4.28818	4.[illegible]
3.21264	3.2042	3.20829	3.22622	3.25956	3.31027	3.38072	3.[illegible]
4.43235	4.03382	3.632	3.22622	2.81561	2.39907	1.97519	1.[illegible]
4.45291	4.38847	4.33262	4.28647	4.25134	4.22888	4.22105	4.[illegible]
2.79134	2.78552	2.79314	2.81561	2.85456	2.91198	2.99022	3.[illegible]
4.45291	4.05814	3.66054	3.25956	2.85456	2.44474	2.02923	1.[illegible]
4.48839	4.41058	4.34061	4.2796	4.22888	4.19006	4.07593	3.[illegible]
2.36257	2.3596	2.37127	2.39907	2.44474	2.51032	2.61055	2.[illegible]
4.48839	4.09811	3.70552	3.31027	2.91198	2.51032	2.11341	1.[illegible]
4.54102	4.44941	4.36476	4.28818	4.22105	4.07593	3.78223	3.[illegible]
1.92437	1.92453	1.94092	1.97519	2.02923	2.11341	2.23442	2.[illegible]
4.54102	4.15604	3.76932	3.38072	2.99022	2.61055	2.23442	1.[illegible]
4.61369	4.50785	4.40782	4.31477	4.14206	3.82714	3.51219	3.[illegible]
1.47402	1.47762	1.49969	1.54215	1.61353	1.70744	1.82396	1.[illegible]
4.61369	4.23496	3.855	3.4739	3.11355	2.76394	2.39038	1.[illegible]
4.71028	4.58976	4.47219	4.27491	3.96139	3.63883	3.30627	2.[illegible]
1.00763	1.01504	1.04444	1.10183	1.16969	1.25195	1.3547	1.[illegible]
4.71028	4.33894	3.96702	3.63113	3.30877	2.97063	2.60996	2.[illegible]
4.83631	4.7456	4.45487	4.18475	3.88582	3.57782	3.25884	3.[illegible]
0.519366	0.537691	0.567417	0.606472	0.652603	0.708503	0.776708	0.[illegible]
4.83631	4.46592	4.17717	3.88222	3.58336	3.27425	2.95241	2.[illegible]
4.91278	4.73885	4.46647	4.19235	3.91246	3.62714	3.33576	3.[illegible]
0.264343	0.280284	0.299077	0.321825	0.349191	0.382173	0.421651	0.[illegible]
4.91278	4.5906	4.32099	4.04697	3.76843	3.48446	3.19468	3.[illegible]
5.	4.75	4.5	4.25	4.	3.75	3.5	3.[illegible]
0	0	0	0	0	0	0	0
5.	4.75	4.5	4.25	4.	3.75	3.5	3.[illegible]

In[111]:= fulltablefight := Table[tableentry[i, j, halftablefight1234], {i, 12}, {j, 12}];

In[112]:= fulltablefight // TableForm

Out[112]//TableForm=

5.55556<br>5.55556<br>5.55556	5.5137<br>5.5137<br>5.07558	5.46994<br>5.46994<br>4.58224	5.42404<br>5.42404<br>4.07458	5.37569<br>5.37569<br>3.55147	5.3245<br>5.3245<br>3.01164	5.26997<br>5.26997<br>2.45363	5.2<br>5.2<br>1.8
5.5137<br>5.07558<br>5.5137	5.46683<br>5.04007<br>5.04007	5.41753<br>5.00318<br>4.55282	5.36539<br>4.96478<br>4.05091	5.30995<br>4.92474<br>3.53318	5.25056<br>4.88289<br>2.99827	5.18633<br>4.83908<br>2.44461	5.1<br>4.7<br>1.8
5.46994<br>4.58224<br>5.46994	5.41753<br>4.55282<br>5.00318	5.36206<br>4.5225<br>4.5225	5.30302<br>4.49127<br>4.02679	5.23973<br>4.45915<br>3.51481	5.17124<br>4.42624<br>2.98511	5.09626<br>4.39273<br>2.43601	5.0<br>4.3<br>1.8
5.42404<br>4.07458<br>5.42404	5.36539<br>4.05091<br>4.96478	5.30302<br>4.02679<br>4.49127	5.23625<br>4.00231<br>4.00231	5.16414<br>3.97766<br>3.49656	5.08545<br>3.95315<br>2.97246	4.99839<br>3.92937<br>2.42815	4.9<br>3.9<br>1.8
5.37569<br>3.55147<br>5.37569	5.30995<br>3.53318<br>4.92474	5.23973<br>3.51481<br>4.45915	5.16414<br>3.49656<br>3.97766	5.082<br>3.47877<br>3.47877	4.99167<br>3.46197<br>2.96075	4.8908<br>3.44712<br>2.42157	4.7<br>3.5<br>1.8
5.3245<br>3.01164<br>5.3245	5.25056<br>2.99827<br>4.88289	5.17124<br>2.98511<br>4.42624	5.08545<br>2.97246<br>3.95315	4.99167<br>2.96075<br>3.46197	4.88781<br>2.95075<br>2.95075	4.72396<br>3.00933<br>2.46682	4.4<br>3.1<br>2.0
5.26997<br>2.45363<br>5.26997	5.18633<br>2.44461<br>4.83908	5.09626<br>2.43601<br>4.39273	4.99839<br>2.42815<br>3.92937	4.8908<br>2.42157<br>3.44712	4.72396<br>2.46682<br>3.00933	4.46884<br>2.64004<br>2.64004	4.2<br>2.8<br>2.2
5.21144<br>1.87573<br>5.21144	5.11603<br>1.87039<br>4.79316	5.0129<br>1.86553<br>4.35906	4.90033<br>1.86149<br>3.90737	4.72708<br>1.89043<br>3.51335	4.47137<br>2.03119<br>3.19892	4.21435<br>2.20742<br>2.8517	3.9<br>2.4<br>2.4
5.14795<br>1.27591<br>5.14795	5.03779<br>1.27341<br>4.74506	4.91746<br>1.27161<br>4.32728	4.73297<br>1.28469<br>3.97446	4.47846<br>1.38241<br>3.69927	4.22258<br>1.50277<br>3.41323	3.96466<br>1.65721<br>3.10721	3.7<br>1.8<br>2.7
5.07816<br>0.651691<br>5.07816	4.98055<br>0.614309<br>4.65592	4.72689<br>0.666308<br>4.40734	4.48869<br>0.703085<br>4.13478	4.23597<br>0.761739<br>3.88051	3.98275<br>0.831577<br>3.6251	3.72911<br>0.915139<br>3.36779	3.4<br>1.<br>3.
5.0403<br>0.329504<br>5.0403	4.87088<br>0.318443<br>4.70094	4.61916<br>0.342011<br>4.4523	4.36879<br>0.368047<br>4.20202	4.11762<br>0.398782<br>3.95351	3.86664<br>0.433592<br>3.706	3.61634<br>0.471733<br>3.45984	3.3<br>0.3<br>3.
5.<br>0<br>5.	4.75<br>0<br>4.75	4.5<br>0<br>4.5	4.25<br>0<br>4.25	4.<br>0<br>4.	3.75<br>0<br>3.75	3.5<br>0<br>3.5	3.2<br>0<br>3.

In[113]:= (* We use ListInterpolation to create continuous functions. payofffn1 gives income of the strongest player with resources of 10 when facing two weaker players. *)

In[114]:= payofffn1 = ListInterpolation[fulltablepayoff[[All, All, 1]],
{{10, 9, 8, 7, 6, 5, 4, 3, 2, 1, 0.5, 0}, {10, 9, 8, 7, 6, 5, 4, 3, 2, 1, 0.5, 0}}]

Out[114]= InterpolatingFunction[Domain: {{0., 10.}, {0., 10.}} Output: scalar]

In[115]:= (* payofffn2 and payofffn3 give incomes of the weaker players when facing a strong player with resources of 10 and another weak player *)

In[116]:= payofffn2 = ListInterpolation[fulltablepayoff[[All, All, 2]],
{{10, 9, 8, 7, 6, 5, 4, 3, 2, 1, 0.5, 0}, {10, 9, 8, 7, 6, 5, 4, 3, 2, 1, 0.5, 0}}]

Out[116]= InterpolatingFunction[Domain: {{0., 10.}, {0., 10.}} Output: scalar]

In[117]:=
```
payofffn3 = ListInterpolation[fulltablepayoff[[All, All, 3]],
   {{10, 9, 8, 7, 6, 5, 4, 3, 2, 1, 0.5, 0}, {10, 9, 8, 7, 6, 5, 4, 3, 2, 1, 0.5, 0}}]
```

Out[117]=
```
InterpolatingFunction[ Domain: {{0., 10.}, {0., 10.}}
                       Output: scalar ]
```

In[118]:=
```
(* A contour plot of payoff to the stronger player *)
```

In[119]:=
```
(* FIGURE 2a *)
```

In[120]:=
```
Show[ContourPlot[payofffn1[y, z], {y, 0, 10}, {z, 0, 10},
   Contours → {5.6, 5.4, 5.2, 5, 4.8, 4.6, 4.4, 4.2, 4, 3.8, 3.6, 3.4, 3.2, 3, 2.8},
   ContourLabels → True, ColorFunction → GrayLevel,
   ColorFunctionScaling → {0, 1}], FrameLabel →
   {{HoldForm[player 3 resources], None}, {HoldForm[player 2 resources], None}},
  PlotLabel → None, LabelStyle → {GrayLevel[0]}]
```

Out[120]=

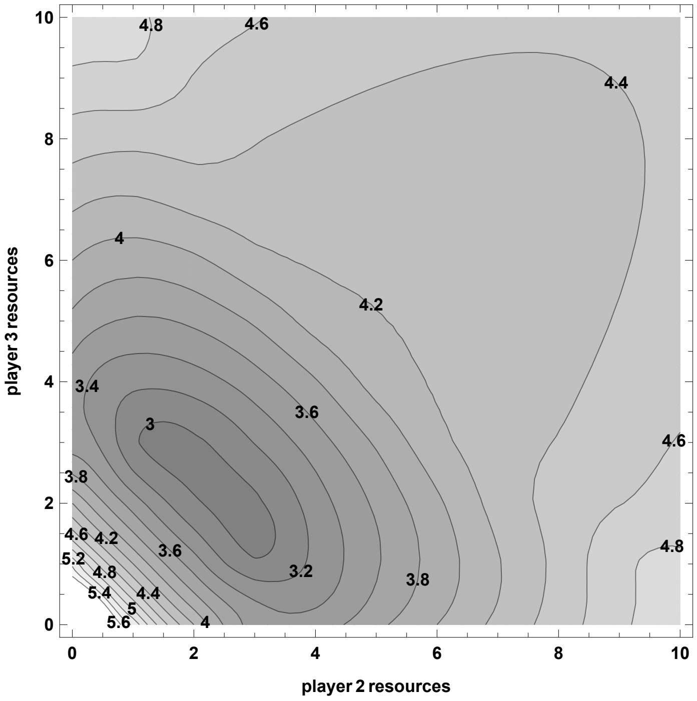

In[121]:=
```
(* A 3D plot of payoff to the stronger player *)
```

In[122]:=
```
Plot3D[payofffn1[y, z], {y, 0, 10}, {z, 0, 10}, PlotRange → {0, 6}]
```

Out[122]=

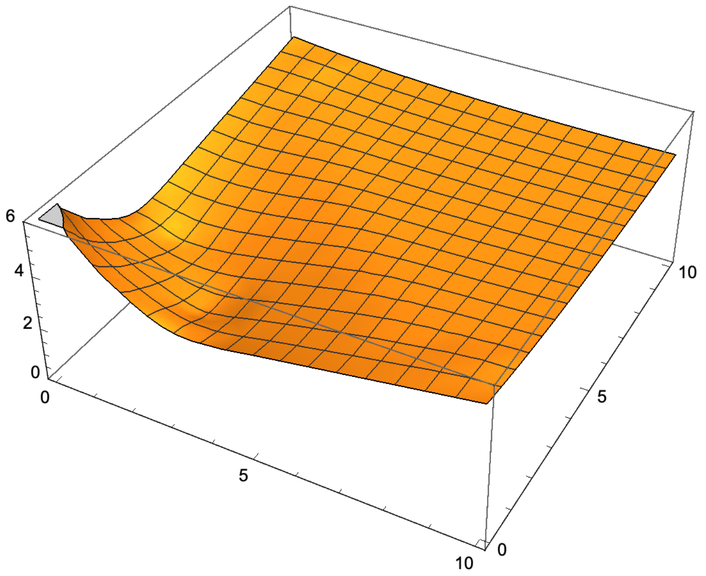

In[123]:=
```
(* A contour plot of payoff to one of the weaker players - player 2 *)
```

In[124]:=
```
(* FIGURE 2b *)
```

In[125]:=
```
Show[ContourPlot[payofffn2[y, z], {y, 0, 10}, {z, 0, 10},
  Contours → {5, 4.8, 4.6, 4.4, 4.2, 4, 3.8, 3.6, 3.4, 3.2, 3, 2.8, 2.6, 2.2,
    2.4, 2.2, 2, 1.8, 1.6, 1.4, 1.2, 1, .8, .6, .4, .2}, ContourLabels → True,
  ColorFunction → GrayLevel, ColorFunctionScaling → {.5, 1}], FrameLabel →
  {{HoldForm[player 3 resources], None}, {HoldForm[player 2 resources], None}},
 PlotLabel → None, LabelStyle → {GrayLevel[0]}]
```

Out[125]=

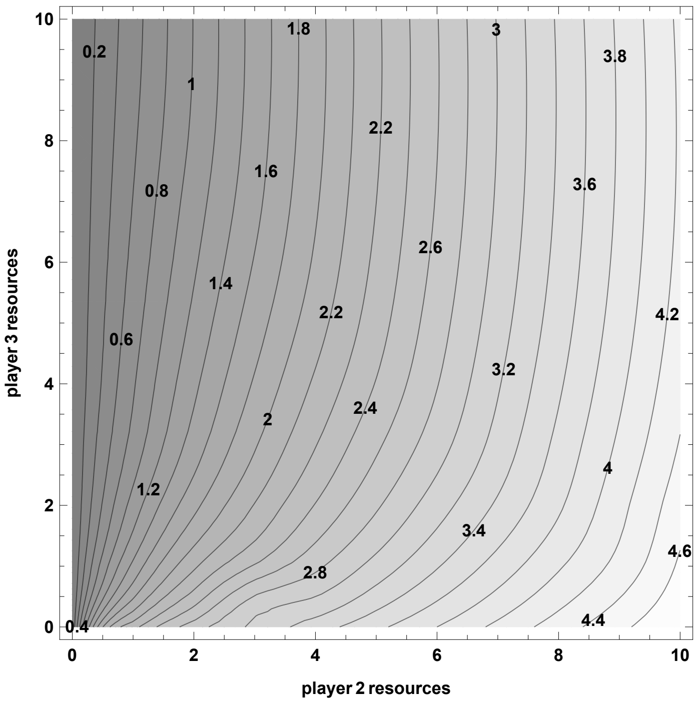

In[126]:=
```
(* A contour plot of payoff to the other weaker player - player 3*)
```

In[127]:=
```
Show[ContourPlot[payofffn3[y, z], {y, 0, 10}, {z, 0, 10},
  Contours → {5, 4.8, 4.6, 4.4, 4.2, 4, 3.8, 3.6, 3.4, 3.2, 3, 2.8, 2.6, 2.2,
    2.4, 2.2, 2, 1.8, 1.6, 1.4, 1.2, 1, .8, .6, .4, .2}, ContourLabels → True,
  ColorFunction → GrayLevel, ColorFunctionScaling → {.5, 1}], FrameLabel →
  {{HoldForm[player 3 resources], None}, {HoldForm[player 2 resources], None}},
 PlotLabel → None, LabelStyle → {GrayLevel[0]}]
```

Out[127]=

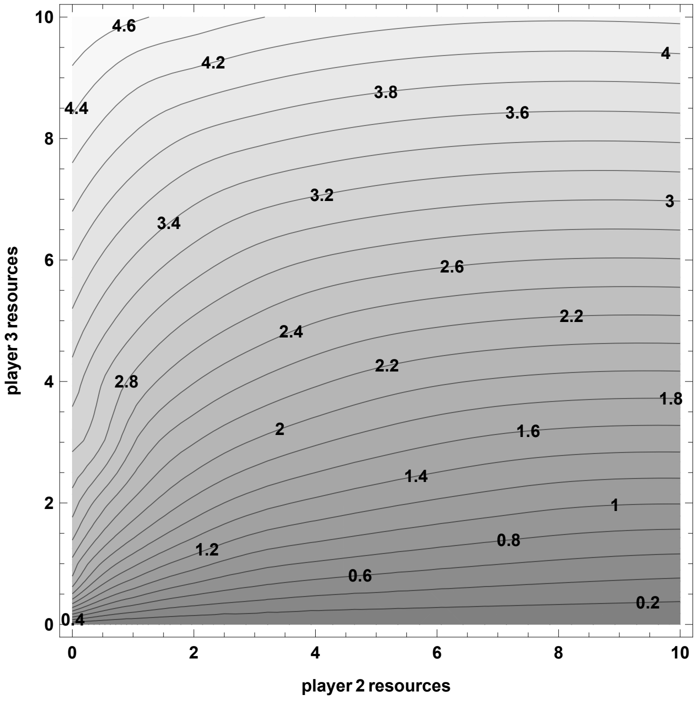

In[128]:=
```
(* A 3-D plot of payoff to one of the weaker players *)
```

In[129]:=
```
Plot3D[payofffn2[y, z], {y, 0, 10}, {z, 0, 10}, PlotRange → {0, 6}]
```

Out[129]=

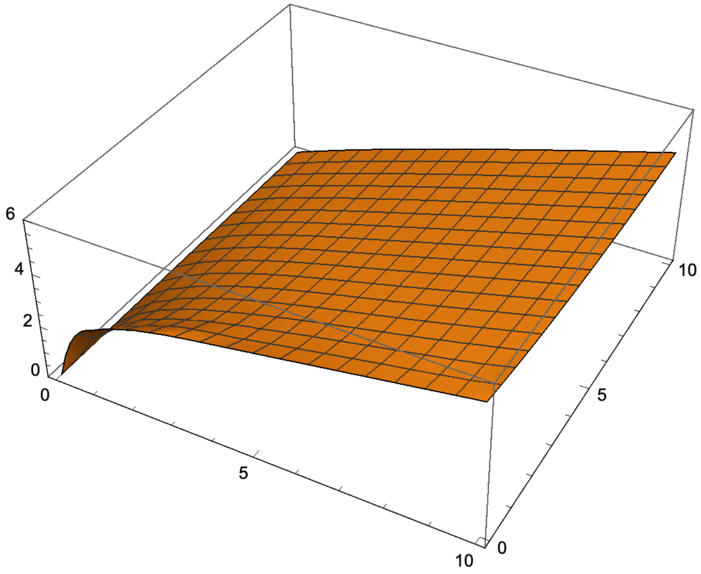

In[130]:= (* A 3-D plot of payoff to the other weaker player *)

```
In[131]:= Plot3D[payofffn3[y, z], {y, 0, 10}, {z, 0, 10}, PlotRange → {0, 6}, AxesLabel → {x, y}]
```

Out[131]=

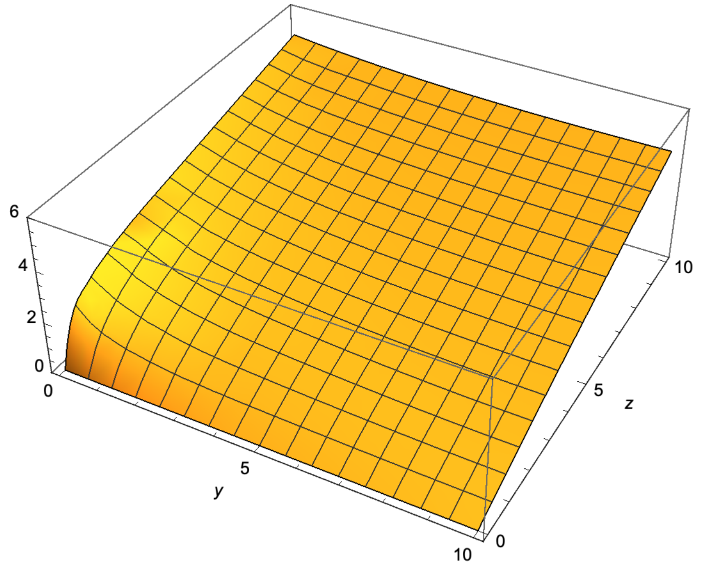

In[132]:= (* We use ListInterpolation to create continuous functions. fightfn1 gives total fighting effort of the strongest player with resources of 10 when facing two weaker players. *)

```
In[133]:= fightfn1 = ListInterpolation[fulltablefight[[All, All, 1]],
  {{10, 9, 8, 7, 6, 5, 4, 3, 2, 1, 0.5, 0}, {10, 9, 8, 7, 6, 5, 4, 3, 2, 1, 0.5, 0}}]
```

Out[133]= InterpolatingFunction[Domain: {{0., 10.}, {0., 10.}} Output: scalar]

In[134]:= (* fightfn2 and fightfn3 give total fighting effort of the weaker players when facing a strong player with resources of 10 and another weak player *)

```
In[135]:= fightfn2 = ListInterpolation[fulltablefight[[All, All, 2]],
  {{10, 9, 8, 7, 6, 5, 4, 3, 2, 1, 0.5, 0}, {10, 9, 8, 7, 6, 5, 4, 3, 2, 1, 0.5, 0}}]
```

Out[135]= InterpolatingFunction[Domain: {{0., 10.}, {0., 10.}} Output: scalar]

```
In[136]:= fightfn3 = ListInterpolation[fulltablefight[[All, All, 3]],
  {{10, 9, 8, 7, 6, 5, 4, 3, 2, 1, 0.5, 0}, {10, 9, 8, 7, 6, 5, 4, 3, 2, 1, 0.5, 0}}]
```

Out[136]= InterpolatingFunction[Domain: {{0., 10.}, {0., 10.}} Output: scalar]

In[137]:= (* A contour plot of fighting effort of the stronger player *)

```
In[138]:= ContourPlot[
  {fightfn1[y, z] == {5.6, 5.4, 5.2, 5, 4.8, 4.6, 4.4, 4.2, 4, 3.8, 3.6, 3.4, 3.2,
     3, 2.8, 2.6, 2.4, 2.2, 2}}, {y, 0, 10}, {z, 0, 10}, PlotRange → {0, 6}]
```

Out[138]=

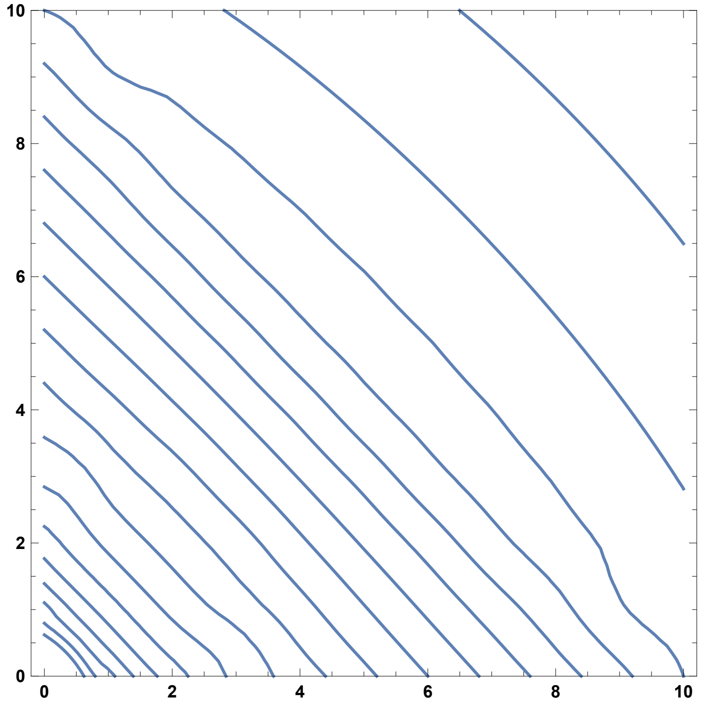

```
In[139]:= (* A 3-D plot of fighting effort of the stronger player *)

In[140]:= Plot3D[fightfn1[y, z], {y, 0, 10}, {z, 0, 10}, PlotRange → {0, 6}]
```

Out[140]=

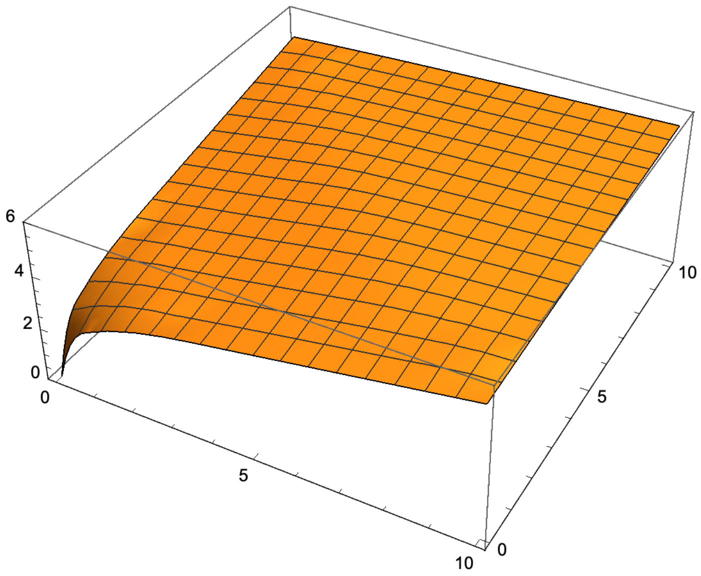

```
In[141]:= (* A contour plot of payoff to one of the weaker players *)
```

In[142]:=
```
ContourPlot[
 {fightfn3[y, z] == {4.8, 4.6, 4.4, 4.2, 4, 3.8, 3.6, 3.4, 3.2, 3, 2.8, 2.6, 2.4, 2.2, 2,
    1.8, 1.6, 1.4, 1.2, 1, .8, .6, .4, .2}}, {y, 0, 10}, {z, 0, 10}, PlotRange → {0, 6}]
```

Out[142]=

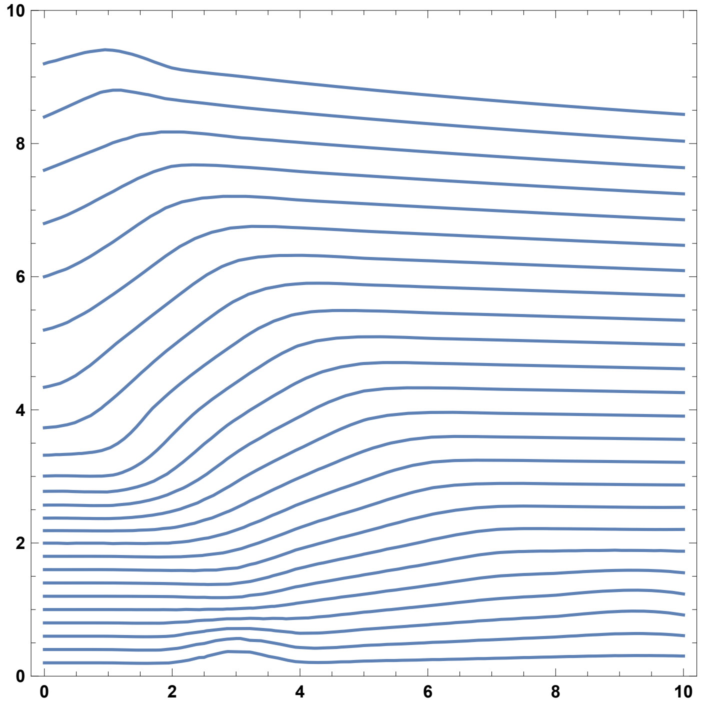

In[143]:=
```
(* A 3-D plot of payoff to one of the weaker players *)
```

In[144]:=
```
Plot3D[fightfn3[y, z], {y, 0, 10}, {z, 0, 10}, PlotRange → {0, 6}]
```

Out[144]=

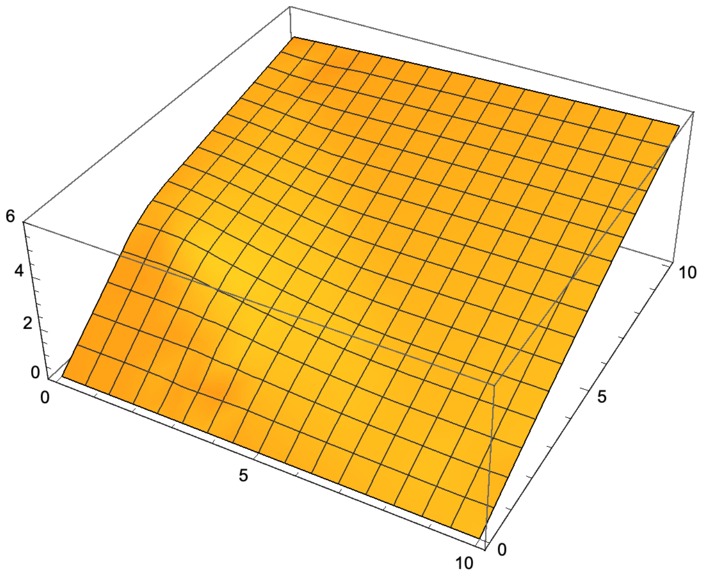

In[145]:=
```
(* We combine all the payofffn functions above to get
 payofffn[x,y,z] which gives the income of x for any {x,y,z} *)
```

In[146]:=
```
Clear[payofffn]
```

In[147]:=
```
payofffn[x_, y_, z_] := (x / 10) payofffn1[y * 10 / x, z * 10 / x] /; x >= y && x >= z
```

In[148]:=
```
payofffn[x_, y_, z_] := (z / 10) payofffn2[x * 10 / z, y * 10 / z] /; z >= y && z ≥ x
```

In[149]:=
```
payofffn[x_, y_, z_] := (y / 10) payofffn2[x * 10 / y, z * 10 / y] /; y ≥ z && y ≥ x
```

In[150]:=
```
(* We combine the disjoint parts of payofffn[x,y,z] to get a smooth function *)
```

In[151]:=
```
payoffinterpolate = FunctionInterpolation[payofffn[x, y, z],
  {x, 0.0000001, 10}, {y, 0.0000001, 10}, {z, 0.0000001, 10}]
```

Out[151]=
```
InterpolatingFunction[ Domain: {{1.×10^-7, 10.}, {1.×10^-7, 10.}, {1.×10^-7, 10.}}
                       Output: scalar ]
```

In[152]:=
```
(* We combine all the fightfn functions above to get
 fightfn[x,y,z] which gives the fighting effort of x for any {x,y,z} *)
```

In[153]:=
```
Clear[fightfn]
```

In[154]:=
```
fightfn[x_, y_, z_] := (x / 10) fightfn1[y * 10 / x, z * 10 / x] /; x >= y && x >= z
```

In[155]:=
```
fightfn[x_, y_, z_] := (z / 10) fightfn2[x * 10 / z, y * 10 / z] /; z >= y && z ≥ x
```

In[156]:=
```
fightfn[x_, y_, z_] := (y / 10) fightfn2[x * 10 / y, z * 10 / y] /; y ≥ z && y ≥ x
```

In[157]:=
```
(* We combine the disjoint parts of payofffn[x,y,z] to get a smooth function *)
```

In[158]:=
```
fightinterpolate = FunctionInterpolation[fightfn[x, y, z],
  {x, 0.0000001, 10}, {y, 0.0000001, 10}, {z, 0.0000001, 10}]
```

Out[158]=
```
InterpolatingFunction[ Domain: {{1.×10^-7, 10.}, {1.×10^-7, 10.}, {1.×10^-7, 10.}}
                       Output: scalar ]
```

In[159]:=
```
(* PART THREE. A game in space *)
```

In[160]:=
```
(* For each player i we calculate average income and
  fighting effort over all three player games among i and j and k -
 assuming 20 players in a line in space. *)
```

In[161]:=
```
Clear[nslice, w, p, initialresourcelist]
```

In[162]:=
```
nslice := 20
```

In[163]:=
```
(* The variable distanceindex[i] lets us normalize our weighted average *)
```

In[164]:=
```
distanceindex[ii_] :=
 Total[Flatten[Table[(1 - Sqrt[(ii - j)^2 / 2 + (ii - k)^2 / 2] / nslice)^2,
    {j, Cases[Table[j, {j, 1, nslice}], Except[ii]]},
    {k, Cases[Table[k, {k, 1, j - 1}], Except[ii]]}]]]
```

In[165]:=
```
(* w[i] is average payoff to player i over all games weighted by distance -
 i.e. games with nearby players count
  for more than games with distant players. *)
```

In[166]:=
```
w[ii_, rvector_] :=
 Total[Flatten[Table[(1 - Sqrt[(ii - j)^2 / 2 + (ii - k)^2 / 2] / nslice)^2 payoffinterpolate[
       rvector[[ii]], rvector[[j]], rvector[[k]]],
     {j, Cases[Table[j, {j, 1, nslice}], Except[ii]]},
     {k, Cases[Table[k, {k, 1, j - 1}], Except[ii]]}]]] / distanceindex[ii]
```

In[167]:=
```
(* p[i] is average payoff to player i over all games weighted by distance -
 i.e. games with nearby players count
  for more than games with distant players. *)
```

In[168]:=
```
p[ii_, rvector_] :=
 Total[Flatten[Table[(1 - Sqrt[(ii - j)^2 / 2 + (ii - k)^2 / 2] / nslice)^2 fightinterpolate[
       rvector[[ii]], rvector[[j]], rvector[[k]]],
     {j, Cases[Table[j, {j, 1, nslice}], Except[ii]]},
     {k, Cases[Table[k, {k, 1, j - 1}], Except[ii]]}]]] / distanceindex[ii]
```

In[169]:=
```
(* wp[ii_,rvector_]:=
 Total[Flatten[Table[(1-Sqrt[(ii-j)^2/2+(ii-k)^2/2]/nslice)^2Ramp[
       rvector[[ii]]-fightinterpolate[rvector[[ii]],rvector[[j]],rvector[[k]]]-
        payoffinterpolate[rvector[[ii]],rvector[[j]],rvector[[k]]]-
        .1rvector[[ii]]],{j,Cases[Table[j,{j,1,nslice}],Except[ii]]},
     {k,Cases[Table[k,{k,1,j-1}],Except[ii]]}]]]/distanceindex[ii] *)
```

In[170]:=
```
initialresourcelist = N[Table[E ^ (2.2 - .4 ii), {ii, 1, nslice}]]
```

Out[170]=
```
{6.04965, 4.0552, 2.71828, 1.82212, 1.2214, 0.818731, 0.548812,
 0.367879, 0.246597, 0.165299, 0.110803, 0.0742736, 0.0497871, 0.0333733,
 0.0223708, 0.0149956, 0.0100518, 0.00673795, 0.00451658, 0.00302755}
```

In[171]:=
```
(* We assume resources decline exponentially from center to periphery. *)
```

In[172]:=
```
ListLinePlot[initialresourcelist, PlotRange → All]
```

Out[172]=

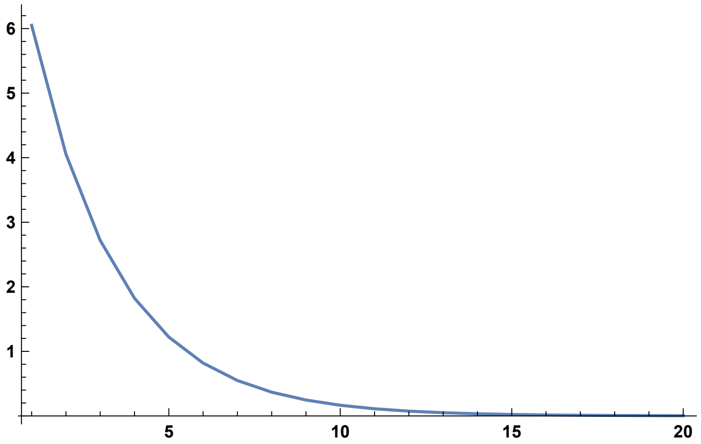

In[173]:=
```
(* Wealth exceeds power at the core. Power exceeds wealth at the periphery. *)
```

In[174]:=
```
ListLinePlot[{initialresourcelist - Table[p[i, initialresourcelist], {i, 1, nslice}],
   Table[w[i, initialresourcelist], {i, 1, nslice}]}, PlotRange → All,
  DataRange → {0, 100}, PlotLabels → {Callout["production", {Scaled[0.08], Above}],
    Callout["income", {Scaled[0.25], Above}]}, AxesLabel → {"distance", "value"}]
```

Out[174]=

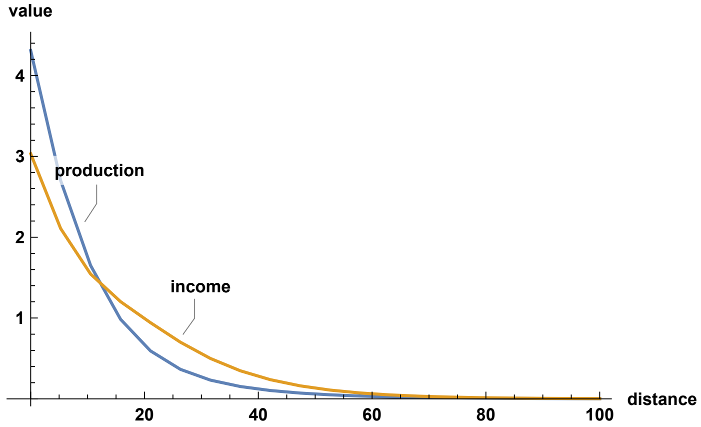

In[175]:=
```
(* PART FOUR. A game in space and time *)
```

In[176]:=
```
Clear[r, a, c, tlag, resourcelist, resourceeqns0,
  resourceeqns1, resourceeqns2, newresourcelist, resourcehistory]
```

In[177]:=
```
(* The vectors resourcelist and resourceeqns0 and
   resourceeqns1 and resourceeqns2 will let us produce a difference-
  equation version of the partial differental equation for changes
   in  resources and wealth and power over space and time. *)
```

In[178]:=
```
resourcelist := Table[ resource[i, t], {i, 1, nslice}]
```

In[179]:=
```
resourceeqns0 := Table[ resource[i, t] (10 - resource[i, t]), {i, 1, nslice}]
```

In[180]:=
```
resourceeqns1 := Append[Table[resource[i + 1, t] - resource[i, t], {i, 0, nslice}], 0]
```

In[181]:=
```
resourceeqns2 := Prepend[Table[resource[i, t] - resource[i - 1, t], {i, 1, nslice + 1}],
   resource[0, t] - resource[1, t]]
```

In[182]:=
```
(* wp[i,r] is the wealth-power mismatch.*)
```

In[183]:=
```
wp[ii_, rvector_] :=
  Total[Flatten[Table[(1 - Sqrt[(ii - j)^2 / 2 + (ii - k)^2 / 2] / nslice)^2 Ramp[
        rvector[[ii]] - fightinterpolate[rvector[[ii]], rvector[[j]], rvector[[k]]] -
         payoffinterpolate[rvector[[ii]], rvector[[j]], rvector[[k]]] -
         .1 rvector[[ii]]], {j, Cases[Table[j, {j, 1, nslice}], Except[ii]]},
      {k, Cases[Table[k, {k, 1, j - 1}], Except[ii]]}]]] / distanceindex[ii]
```

In[184]:=
```
(* Given a resource vector r,
the function newresourcelist[r,tlag] gives resources in the next time interval
  depending on logistic growth and spatial diffusion and collateral damage. *)
```

In[185]:=
```
newresourcelist[oldresourcelist_, tlag_] :=
 oldresourcelist + r oldresourcelist (10 - oldresourcelist) +
  a (Prepend[Drop[oldresourcelist, -1], oldresourcelist[[2]]] - 2 oldresourcelist +
     Append[Drop[oldresourcelist, 1], oldresourcelist[[nslice]]]) -
  c * Table[wp[i, oldresourcelist], {i, 1, nslice}] * LogisticSigmoid[tlag / 6 - 4]
```

In[186]:=
```
(* Here's the results for a particular combnation of of r
 a c and timelag where the function resourcehistory applies the
 newresourcelist function repeatedly to give the evolution over time *)
```

In[187]:=
```
r := .009;
a := .001;
c := 8;
tlag := 5;
```

In[191]:=
```
resourcehistory = FoldList[newresourcelist, initialresourcelist, Range[60]];
```

In[192]:=
```
(* FIGURE 4a *)
```

In[193]:=
```
ListPlot3D[resourcehistory, PlotRange → All, DataRange → {{0, 100}, {0, 1200}, {0, 6}},
 AxesLabel → {"distance", "year CE", "resources"},
 Ticks → {{0, 20, 40, 60, 80, 100}, {{0, "0"}, {200, ""}, {400, "400"},
    {600, ""}, {800, "800"}, {1000, ""}, {1200, "1200"}}, {2, 4, 6, 8, 10}},
 ColorFunction → GrayLevel, ColorFunctionScaling → {.4, 1}]
```

Out[193]=
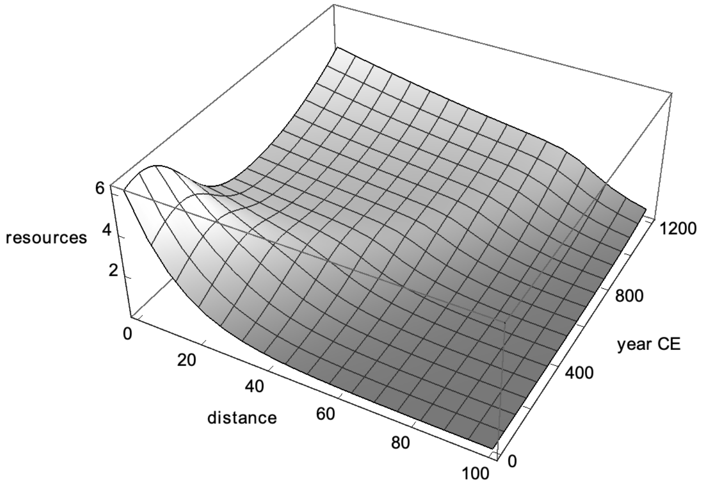

In[194]:=
```
Clear[wlist, plist]
```

In[195]:=
```
wlist[somelist_] := Table[w[i, somelist], {i, 1, 20}]
```

In[196]:=
```
whistory = Map[wlist, resourcehistory];
```

In[197]:=
```
ListPlot3D[whistory]
```

Out[197]=

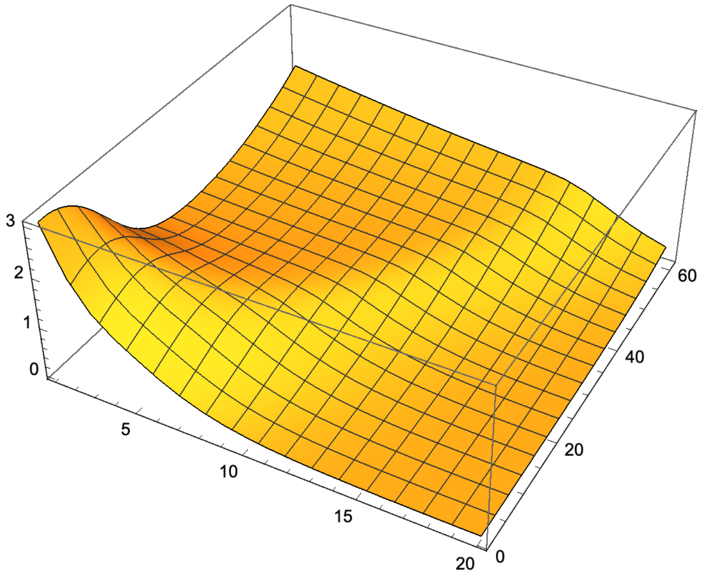

In[198]:=
```
plist[somelist_] := Table[p[i, somelist], {i, 1, 20}];
```

In[199]:=
```
phistory = Map[plist, resourcehistory];
```

In[200]:=
```
ListPlot3D[phistory]
```

Out[200]=

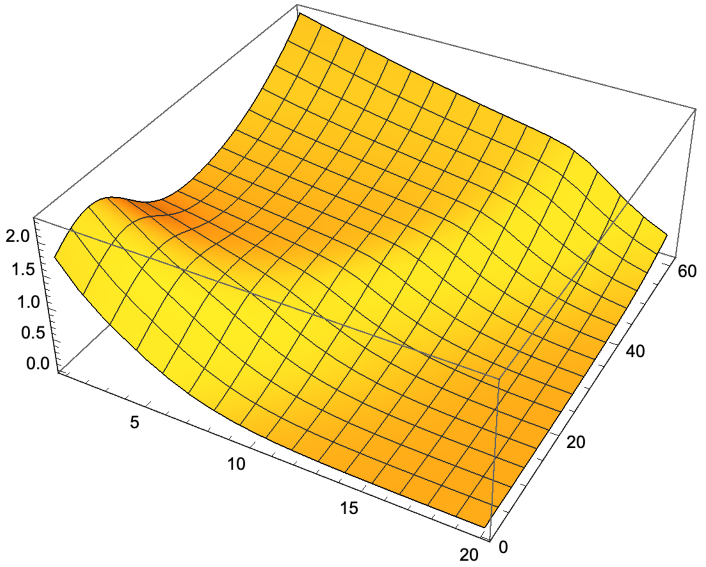

In[201]:=
```
ListPlot3D[{resourcehistory - phistory, whistory},
 PlotRange → All, DataRange → {{0, 100}, {0, 1200}, {0, 6}},
 AxesLabel → {"distance", "year CE", "value"},
 Ticks → {{0, 20, 40, 60, 80, 100}, {{0, "0"}, {200, ""}, {400, "400"},
    {600, ""}, {800, "800"}, {1000, ""}, {1200, "1200"}}, {2, 4, 6, 8, 10}},
 ColorFunction → GrayLevel, ColorFunctionScaling → {.4, 1}]
```

Out[201]=

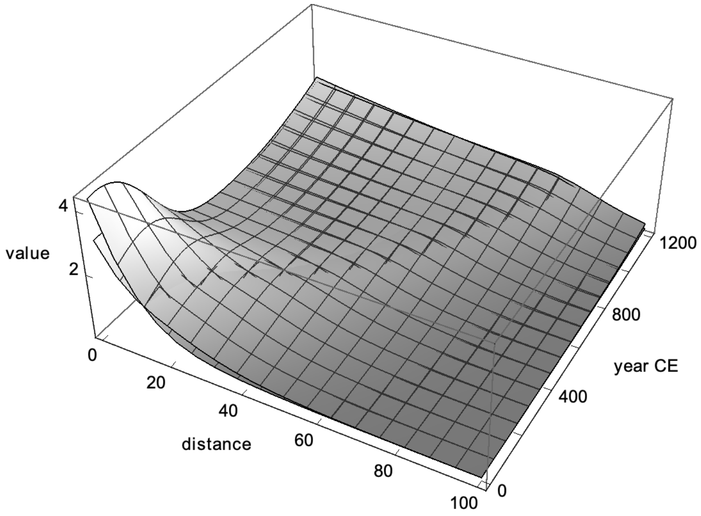

In[202]:=
```
(* FIGURE 4a *)
```

In[203]:=
```
Show[ListPlot3D[resourcehistory - phistory, PlotStyle → {Gray, Opacity[.5]},
  Mesh → None, PlotRange → All, DataRange → {{0, 100}, {0, 1200}, {0, 6}}],
 ListPlot3D[whistory, PlotStyle → Tan, Mesh → 40, PlotRange → All,
  DataRange → {{0, 100}, {0, 1200}, {0, 6}}],
 AxesLabel → {"distance", "year CE", "value"},
 Ticks → {{0, 20, 40, 60, 80, 100}, {{0, "0"}, {200, ""}, {400, "400"},
    {600, ""}, {800, "800"}, {1000, ""}, {1200, "1200"}}, {1, 2, 3, 4}}]
```

Out[203]=

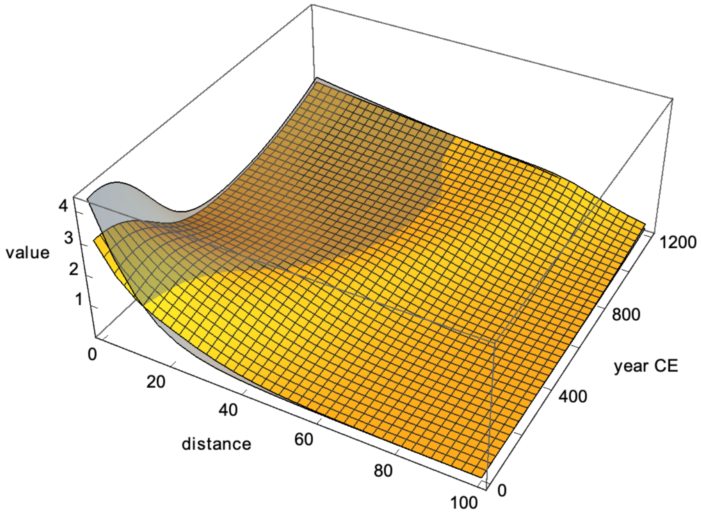

In[204]:= ListLinePlot[{resourcehistory[[1]] - phistory[[1]], whistory[[1]]}, PlotRange → All]

Out[204]=

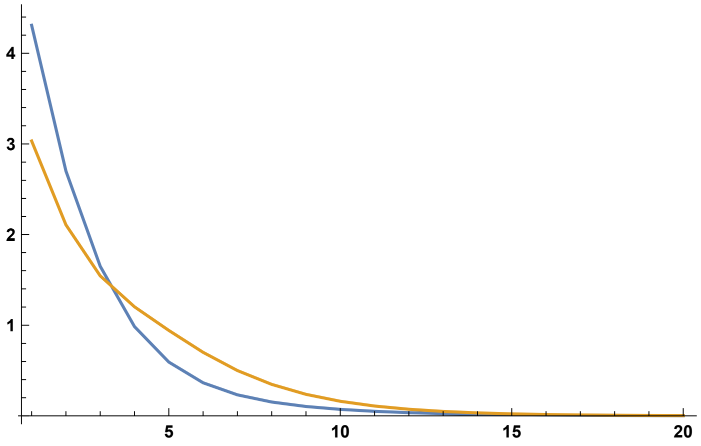

In[205]:= ListLinePlot[
 {resourcehistory[[40]] - phistory[[40]], whistory[[40]]}, PlotRange → All]

Out[205]=

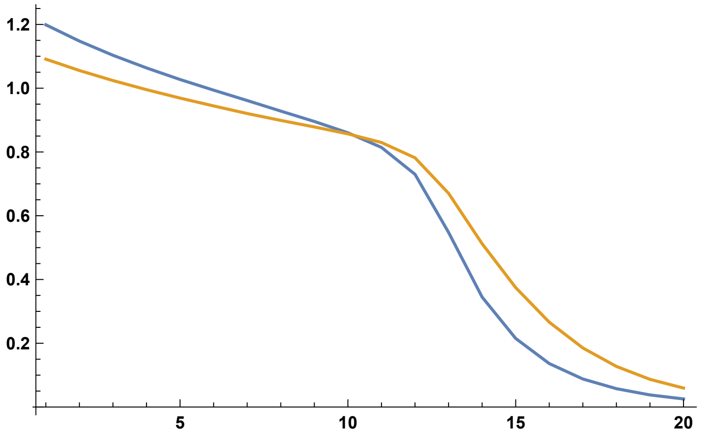

Supplement: S4 File — (PDF) [file pone.0254240.s004.pdf]
